# Salicylic acid improves chilling tolerance via CsNPR1–CsICE1 interaction in grafted cucumbers

**DOI:** 10.1093/hr/uhae231

**Published:** 2024-08-09

**Authors:** Xin Fu, Yiqing Feng, Yanyan Zhang, Huangai Bi, Xizhen Ai

**Affiliations:** Key Laboratory of Crop Biology and Genetic Improvement of Horticultural Crops in Huanghuai Region, College of Horticulture Science and Engineering, Shandong Agricultural University, Tai’an, Shandong 271018, China; Key Laboratory of Crop Biology and Genetic Improvement of Horticultural Crops in Huanghuai Region, College of Horticulture Science and Engineering, Shandong Agricultural University, Tai’an, Shandong 271018, China; Key Laboratory of Crop Biology and Genetic Improvement of Horticultural Crops in Huanghuai Region, College of Horticulture Science and Engineering, Shandong Agricultural University, Tai’an, Shandong 271018, China; Institute of Peanut, Tai’an Academy of Agricultural Sciences, Tai’an, Shandong 271000, China; Key Laboratory of Crop Biology and Genetic Improvement of Horticultural Crops in Huanghuai Region, College of Horticulture Science and Engineering, Shandong Agricultural University, Tai’an, Shandong 271018, China; Key Laboratory of Crop Biology and Genetic Improvement of Horticultural Crops in Huanghuai Region, College of Horticulture Science and Engineering, Shandong Agricultural University, Tai’an, Shandong 271018, China

## Abstract

Salicylic acid (SA) plays a role in the regulation of grafting-induced cold tolerance. However, the molecular mechanism behind it is still unknown. Here, we established that the phenylalanine ammonia-lyase (PAL) pathway-dependent elevate in SA content in grafted cucumber leaves was not only synthesized in the leaves but also transported from the roots under chilling stress. RNAi-*CsPAL* with low SA content as rootstock reduced SA accumulation in grafted seedling leaves while decreasing rootstock-induced cold tolerance, as evidenced by higher electrolyte leakage (EL), hydrogen peroxide (H_2_O_2_), and superoxide anion (O_2_^·−^) contents and lower expression of cold-responsive genes (*CsICE1*, Cs*DREB1A*, *CsDREB1B*, and *CsCOR47*), whereas OE-*CsPAL* with high SA content as rootstock improved the cold tolerance of grafted plants in comparison with the wild type (WT). In addition, *CsNPR1* was significantly upregulated in grafted cucumber under chilling stress, with exogenous and endogenous overexpressed SA inducing its transcriptional expression and protein stability, which exhibited higher expression in grafted plants than in self-root plants. While *CsNPR1*-overexpression (*OE*-*CsNPR1*) seedlings as scions were more tolerant to chilling stress than WT seedlings, *CsNPR1*-suppression (*Anti*-*CsNPR1*) seedlings as scions were more vulnerable to chilling stress. Notably, CsNPR1–CsICE1 interactions alleviated ROS accumulation and activated the expression of *CsDREB1A, CsDREB1B, CsCOR47, CsCOR15, CsCOR413,* and *CsKIN1* to enhance SA-mediated chilling tolerance in grafted cucumber. Overall, our findings reveal that SA enhances chilling tolerance in grafted cucumbers via the model of the CsNPR1–CsICE1 transcriptional regulatory cascade.

## Introduction

Cucumber (*Cucumis sativus* L.), a globally cultivated crop species, exhibits a significant susceptibility to cold stress. In the northern region of China, cucumbers are primarily grown in solar greenhouses during the winter season, but these plants are frequently subjected to chilling stress, which has a significant impact on their growth, productivity, and quality during this period. Grafting is the simplest and most effective method for improving plant resistance to both biotic and abiotic stresses [1] and has been widely used in horticulture crops. Grafting using strong-resistant rootstocks can improve plant tolerance to abiotic stress; for example, heat, cold, drought, and salt [2–5]. Additionally, these rootstocks can enhance the development of above-ground organs by transporting water, mineral salts, and hormones to scions, which then transmit photosynthetic products to rootstocks to promote root growth [6], indicating that a signal interaction exists between rootstock and scion [7].

As the major regulatory component of cold acclimation, the inducer of CBF expression 1 (ICE1)–c-repeat binding factor (CBF)–cold-responsive gene (COR) significantly contributes to the cold stress signaling response pathway [8–11]. It is thought that CBFs are essential for plants in response to cold signal, and it has been found that CBF1, CBF2, and CBF3 [12], which can greatly improve the resistance to cold of overexpression-Arabidopsis seedlings [13–15]. In the ICE1–CBF–COR cascade, ICE1 is an upstream transcription factor (TF) that can trigger the expression of *CBF3* and enhance the cold tolerance [16] and stimulates the expression of CBFs and COR genes by identifying specifically the MYC elements in the CBF promoter [17]. Many components can regulate ICE1 activity. For instance, open stomata 1 (OST1) can phosphorylate ICE1 directly, and it can also bind competitively with high expression of osmotically responsive gene 1 (HOS1) to inhibit the ubiquitination degradation of ICE1, thereby maintaining ICE1 stability and regulating downstream genes expression [18, 19]. Agarwal *et al.* [20] reported that the MYB15 protein cooperates with ICE1 and binds to the MYB recognition site located at the *CBF* gene promoter to control *CBF* expression in response to cold stress. An *et al.* [21] demonstrated that MdABI4–MdICE1 interactions improved the transcriptional regulatory function of MdICE1 on *MdCBF1*, hence promoting the abscisic acid-induced cold tolerance of apples. Moreover, ICE1 transcriptional activity is inhibited through interaction with the inhibitors of jasmonic acid (JA) signaling, that include JAZ1 and JAZ4 [22].

Salicylic acid (SA), as a vital signal molecule, mediates complex biological functions in plants. Plant defense response, particularly the systemic acquired resistance (SAR) as well as hypersensitive response (HR), have been extensively researched in relation to SA [23–25]. Phenylalanine ammonlyase (PAL) as well as isochorismate synthetase (ICS) pathways are two synthetic pathways of SA in higher plants [26] and the PAL pathway is considered to be the main pathway of SA synthesis in cucumber under chilling stress [27]. Phenylalanine ammonlyase is a key rate-limiting enzyme in the PAL pathway and plays a crucial role in the synthesis of SA in response to low temperature [28, 29]. Many studies have established that SA can improve plant tolerance in response to abiotic stresses; for example salt, drought, and high or low temperature [30–33]. While SA plays a role in the communication between rootstock and scion, long-distance transport of SA from rootstock to scion can improve cucumber leaf cold tolerance [34]. Nevertheless, the potential mechanism of SA involvement in rootstock–scion interaction as well as the pathway for SA signal transport remain unclarified.

The SA receptor known as the non-expressor of pathogenesis-related gene 1 (NPR1) includes the BTB/POZ (broad-complex, tramtrack, and bric-a-brac/poxvirus and zinc finger) domain, ankyrin repeat domain (ANK), and NPR1-like structure [35]. In normal circumstances, NPR1 usually localizes as a polymer in the plant cytoplasm, but stress conditions can cause rapid SA accumulation, resulting in polymeric NPR1 being reduced to monomer and transported to the plant nucleus. NPR1 can regulate pathogens-plant response through a transcriptional cascade that is promoted by SA and TGA TFs [36]. Furthermore, SA enhanced the interactions among NPR1, CDK8 (cyclin-dependent kinase 8), and WRKY18 (WRKY DNA-binding protein 18) in Arabidopsis. NPR1 improved its own and target gene levels to enhance plant immunity by recruiting WRKY18, CDK8, and TGA TFs, as well as RNA polymerase II [67, 71]. Notably, Olate *et al.* [37] revealed that NPR1 improved the Arabidopsis cold tolerance via the independent upregulation of cold-induced genes, not mediated by SA and TGA factors. NPR1 interaction with heat shock transcription factor 1 (HSFA1) was also found to stimulate the HSFA1-regulated genes expression and, consequently, plant cold tolerance. NPR1 has been proven to serve as a centralized point that synergistically regulates SA and cold signals [38].

Despite the importance of NPR1 in plant immunity and cold response having been well established, whether its participation in the SA involvement in grafting-induced cold tolerance as well as the underlying regulatory mechanism of it remain understudied. In this study, we present molecular results demonstrating the involvement of SA in the communication between rootstock and scion, and that *CsNPR1* expression in cucumber scion can be encouraged by pumpkin rootstock. While establishing that *CsNPR1* overexpression improves the chilling tolerance of grafted cucumber, we also demonstrate that the CsNPR1-CsICE1 interaction positively regulates grafted cucumber chilling tolerance. This study uncovers a new SA signal regulatory pathway for grafting-induced cold tolerance in plants, which is mediated by NPR1 and cold stress regulatory protein CsICE1.

## Results

### SA improved the grafting-induced cold tolerance of cucumbers

In our earlier studies, it was shown that using pumpkin as a rootstock enhanced the cold tolerance of cucumber and the application of 1 mM exogenous SA enhanced the resistance of grafted cucumber plants to cold stress [34, 39]. Here, we found that *Cs*/*Cm* plants exhibited a cold-damage mitigation effect, significantly lower chilling injury index (CI), electrolyte leakage (EL), and malondialdehyde (MDA) accumulation, and strikingly higher mRNA levels of *CsDREB1A* and *CsICE1* in comparison to *Cs/Cs* plants under cold stress (Fig. S1, see online supplementary material), but at optimum growth temperature (25°C/18°C), most of these indices did not differ. Exogenous SA mitigated the chilling damage and Pn reduction caused by cold stress while upregulating the chilling-induced mRNA levels of the carbon assimilation key genes (*CsRCA, CsrbcL*) and cold-responsive genes (*CsICE1*, *CsDREB1A*, *CsCOR47*) in cucumbers, especially in the *Cs/Cm* plants (*P* < 0.05). However, the SA synthesis inhibitor of L-a-aminooxy-phenylpropionic acid (AOPP) treatment resulted in cold-damage as well as reduced net photosynthetic rate (Pn) and expression of carbon assimilation-related genes and cold-related genes compared to H_2_O treatment under cold stress (Fig. S2, see online supplementary material).

**Figure 1:**
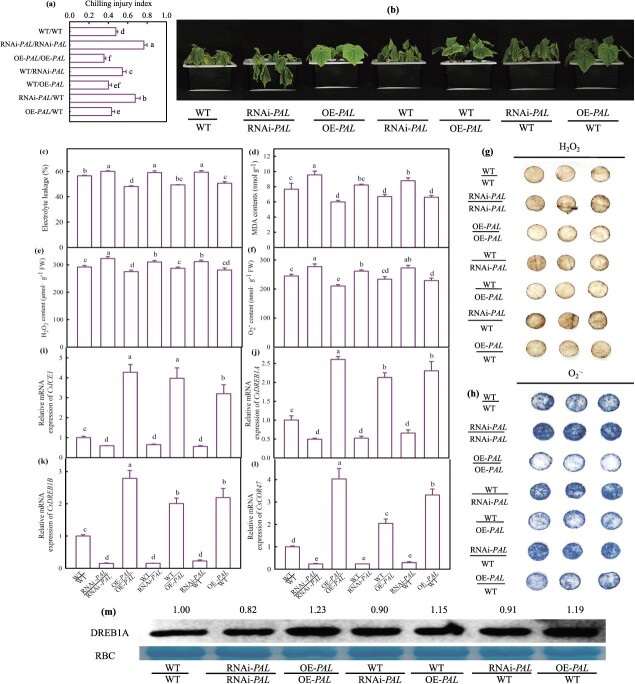
Effect of RNAi and overexpress *CsPAL* on the cold tolerance of self-grafted and grafted cucumbers under cold stress. **(a)** Chilling injury index; **(b)** phenotype of seedlings; **(c–f)** EL, MDA, H_2_O_2_, and O_2_^·-^ content, respectively; **(g, h)** DAB and NBT staining of cucumber leaves. **(i–l)**, Expression of *CsICE1*, *CsDREB1A*, *CsDREB1B*, and *CsCOR47*, respectively; **(m)** DREB1A protein accumulation. The self-grafted and grafted cucumbers plants were treated at 8/5°C for 72 h. Data represent the mean values of four biological replicates, with standard deviations (± SDs) included. With a significance level of *P* < 0.05, different letters differ significantly between samples.

Phenylalanine ammonialyase (PAL) is the key enzyme of salicylic acid synthesis, and *PAL* is the main pathway of salicylic acid synthesis at low temperature [27, 40]. To further verify SA involvement in grafted cucumber chilling tolerance, we obtained overexpression and RNAi transgenic cucumber plants of *CsPAL* (CsaV3_6G039720) using *Agrobacterium-*mediated genetic transformation (Fig. S3, see online supplementary material), and the transgenic lines of OE-*CsPAL*-3 and RNAi-*CsPAL*-92 were chosen for subsequent experiments. At normal temperature (25°C/18°C), the seven grafting combinations of WT/WT, OE-*PAL*/OE-*PAL*, and RNAi-*PAL*/RNAi-*PAL* self-grafted plants, WT grafted onto rootstocks of RNAi-*PAL* and OE-*PAL* transgenic plants (WT/RNAi-*PAL* and WT/OE-*PAL*), as well as RNAi-*PAL* and OE-*PAL* as scion grafted onto rootstocks of WT plants (RNAi-*PAL*/WT and OE-*PAL/*WT) showed no differences (Fig. S4, see online supplementary material), while the RNAi-*PAL*/RNAi-*PAL*, WT/RNAi-*PAL*, and RNAi-*PAL*/WT plants showed severe wilting symptoms and significantly higher CI, EL, and MDA content than the WT/WT plants under chilling stress, the OE-*PAL*/OE-*PAL*, WT/OE-*PAL*, and OE-*PAL/*WT plants markedly alleviated the cold damage and showed lower values (Fig. 1a–d). Compared with the WT/WT plants, the RNAi-*PAL*/RNAi-*PAL*, WT/RNAi-*PAL*, and RNAi-*PAL*/WT plants accumulated more hydrogen peroxide (H_2_O_2_) and superoxide anion (O_2_^.−^), whereas the OE-*PAL*/OE-*PAL*, WT/OE-*PAL*, and OE-*PAL/*WT plants accumulated less under chilling stress. The diaminobenzidine (DAB) and nitroblue tetrazolium (NBT) staining results were consistent with the quantitative results (Fig. 1e–h). Cold stress upregulated the expressions of *CsICE1*, *CsCOR47*, *CsDREB1A*, and *CsDREB1B* in all grafting combinations. Compared with the WT/WT self-grafted plants, the increase of the relative expression of cold responsive genes in RNAi-*PAL*/RNAi-*PAL*, WT/RNAi-*PAL*, and RNAi-*PAL*/WT plants was significantly lower, which was higher in OE-*PAL*/OE-*PAL*, WT/OE-*PAL*, and OE-*PAL/*WT plants after treatment at 8°C/5°C for 72 h (Fig. 1i–l). Under chilling stress, a similar result was observed at the protein level of DREB1A, which was lower in the RNAi-*PAL*/RNAi-*PAL*, WT/RNAi-*PAL*, and RNAi-*PAL*/WT plants but higher in the OE-*PAL*/OE-*PAL*, WT/OE-*PAL*, and OE-*PAL/*WT plants compared to the WT self-grafted plants (Fig. 1m).

### SA participated in rootstock–scion communication of grafted cucumbers

**Figure 2:**
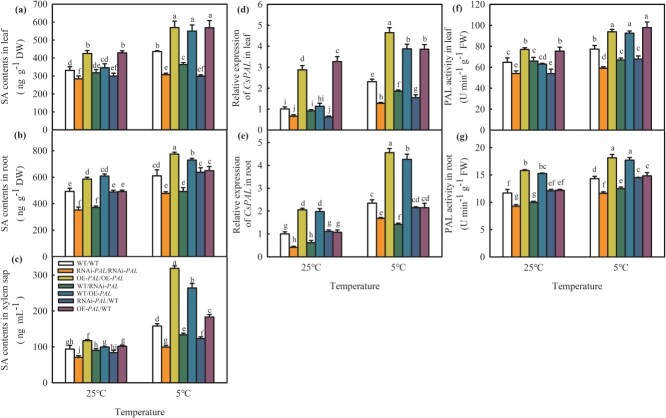
SA is involved in rootstock-scion communication under cold stress. **(a–c)** SA content in leaves, roots and xylem sap, respectively; **(d, e)**  *CsPAL* expression in leaves and roots, respectively; **(f, g)** PAL activity in leaves and roots, respectively. Samples were taken from the third leaf of plants at the three-leaves stage following chilling treatments at 0 h and 12 h. Data represent the mean values of four biological replicates, with standard deviations (± SDs) included. With a significance level of *P* < 0.05, different letters differ significantly between samples.

Compared with the WT/WT plants, SA content and PAL activity in the leaves and roots of RNAi-*PAL*/RNAi-*PAL* transgenic plants were significantly decreased at both room temperature and low temperature, but the OE-*PAL*/OE-*PAL* plants exhibited obviously higher PAL activity and SA content. At normal temperature (25°C), the WT/RNAi-*PAL* and WT/OE-*PAL* leaves as well as the OE-*PAL*/WT and RNAi-*PAL*/WT roots showed similar *CsPAL* mRNA abundance, PAL activity, and SA content to the WT/WT in leaves and roots. However, the *CsPAL* mRNA abundance, PAL activity, and SA content in WT/OE-*PAL* roots and OE-*PAL*/WT leaves significantly exceeded those of WT/WT roots and leaves, but the three parameters in the WT/RNAi-*PAL* roots and RNAi-*PAL*/WT leaves were significantly less than those of WT/WT roots and leaves, respectively. After 12-h exposure at 5°C, the *CsPAL* mRNA abundance, PAL activity, and SA content of all grafting combinations were accumulated significantly, which in the WT/RNAi-*PAL* leaves were 19.9%, 14.4%, and 16.5% lower than those of the WT/WT leaves, respectively. However, these three indices in the WT/OE-*PAL* leaves were 67.5%, 19.8%, and 25.9% higher than those in the WT/WT leaves, respectively (Fig. 2a, d, f). Notably, these three indices markedly decreased in roots of WT/RNAi-*PAL* at both 25°C and 5°C, they strikingly increased in roots of WT/OE-*PAL* (Fig. 2b, e, g), while there is no significant difference among the OE-*PAL*/WT, RNAi-*PAL*/WT, and WT/WT roots. In order to investigate the presence of long-distance transmission of SA between rootstock and scion, we analysed the variation in SA content in xylem sap. As shown in Fig. 2c, the rise of SA content in xylem sap was significantly greater in the OE-*PAL/*OE-*PAL,* WT/OE-*PAL*, and OE-*PAL*/WT plants, while it was noticeably lower in the RNAi-*PAL/*RNAi-*PAL,* WT/RNAi-*PAL*, and RNAi-*PAL*/WT plants than in WT/WT plants. According to these findings, SA signal participates in the rootstock-scion communication of grafted cucumber, which is dependent on the PAL pathway.

### *CsNPR1* participated in *Cm* rootstock-induced cold tolerance

To explore the possible molecular regulated mechanism of SA associated with enhanced cold resistance, we performed transcriptome analysis of *Cs/Cs* and *Cs/Cm* leaves before and after 12 h of being subjected to cold stress (5°C). Among the 912 differentially expressed genes (DEGs) between *Cs/Cs* and *Cs/Cm* plants at 25°C, 459 genes exhibited an upregulation in expression, whereas 453 genes showed a downregulation in expression in *Cs/Cm* leaves. At 5°C, the DEGs add to 6110, include 2774 upregulated genes and 3336 downregulated genes in *Cs/Cm* leaves (Fig. S5a and b, see online supplementary material). Our previous research has analysed the changing level of SA synthesis related genes, such as *PAL*, *ICS*, and *SABP2* in grafted cucumber under cold stress [34], and here we mainly analysed the SA signaling pathway associated genes and found that 9 out of 10 genes were significantly up-regulated (Fig. S6a, see online supplementary material), as determined by the qRT-PCR assays of some key genes of SA-related signal transduction, the relative expression levels of *NPR, TGA*, and *PR* in *Cs/Cm* plants exhibited a considerable increase compared to those in *Cs/Cs* plants at 5°C, corroborating the transcriptome data results. The expression of *NPR* (CsaV3_3G040450; CsaV3_4G007550) in *Cs/Cm* plants response to low temperature was upregulated by 3.15-fold and 2.50-fold, and the expression of *TGA* (CsaV3_3G033620; CsaV3_3G040130) was upregulated by 1.21-fold and 1.37-fold, indicating that *NPR* was more responsive to low temperature in *Cs/Cm* plants (Fig. S5c–e, see online supplementary material). *NPR1* is established as a central element in the signal transduction pathway for SA [41], which serves as the primary factor in this pathway [42] and requires interaction with other TFs to control the downstream genes expression. Previous studies demonstrated that NPR1 could collaborate with TGA to trigger *PRs* expression [43]. More importantly, we also found other up-regulated TFs genes, such as *ICE1*, which was a crucial element of chilling signal transduction pathway, implying that a new *NPR1*-mediated possible signal transduction pathway of SA exists in grafted cucumber under chilling stress. Thus, *NPR1* (CsaV3_4G007550) was chosen as the target gene to be further studied in the next experiment, and we found CsNPR1 protein sequences have conserved BTB/POZ domain, ankyrin repeats, and NPR1/NIM1-like defense protein C-terminal domain, which is consistent with *Arabidopsis thaliana* (Fig. S6b, see online supplementary material).

**Figure 3:**
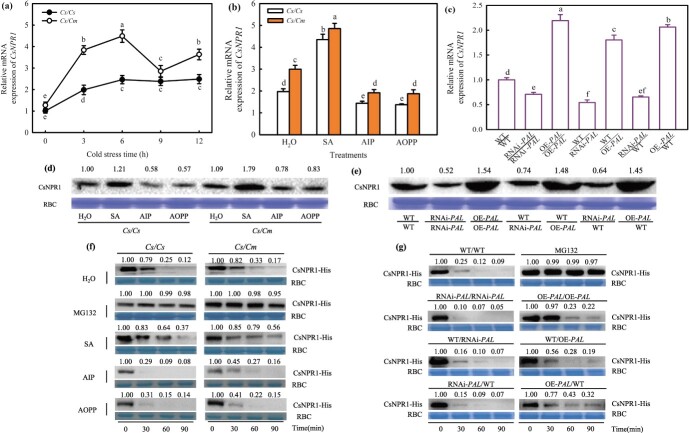
*CsNPR1* participates in cold tolerance induced by SA and grafting. **(a)** Response of *CsNPR1* mRNA abundance to cold stress (5°C) in *Cs/Cs* and *Cs/Cm* leaves. **(b)**  *CsNPR1* expression in *Cs/Cs* and *Cs/Cm* leaves subjected to cold stress as affected by SA, AIP, and AOPP. The three-leaves seedlings were sprayed with 1.0 mM SA, 30 μM AIP, 0.1 mM AOPP, or distilled water (H_2_O, control). Twenty-four hours later, it was exposed to 5°C for 24 h. **(c)**  *CsNPR1* expression in *CsPAL* transgenic self-grafted and grafted cucumbers at 5°C. Data represent the mean values of four biological replicates, with standard deviations (± SDs) included. With a significance level of *P* < 0.05, different letters differ significantly between samples. **(d)** CsNPR1 protein expression in *Cs/Cs* and *Cs/Cm* leaves at 5°C. The seedlings were treated in the same way as in (b). **(e)** CsNPR1 protein in *CsPAL* transgenic self-grafted and grafted cucumbers at 5°C. **(f)** Cell-free degradation experiment of recombinant CsNPR1-HIS protein incubated. The seedlings were pretreated with 1.0 mM SA, 30 μM AIP, 0.1 mM AOPP, or distilled water (H_2_O, control), respectively 24 h before exposure to cold of *Cs/Cs* and *Cs/Cm* plants. **(g)** Cell-free degradation experiment of recombinant CsNPR1-HIS protein in *CsPAL* transgenic self-grafted and grafted cucumbers at 5°C. Then, the proteins were incubated with CsNPR1-HIS for 0, 0.5, 1.0, and 1.5 h. The proteasome inhibitor MG132 was used as control. Anti-His antibody immunoblotting was used to detect the level of proteins in the sample. The protein level at 0 hour was set to 1, and RBC was utilized as actin.

To determine whether *CsNPR1* participates in cold tolerance induced by rootstock, we measured the change of *CsNPR1* mRNA abundances in *Cs/Cs* and *Cs/Cm* leaves exposed to 5°C for 0–12 h. Cold stress significantly induced the mRNA expression levels of *CsNPR1*, with upregulation being most pronounced after 6 h of treatment. The *Cs/Cm* leaves exhibited higher *CsNPR1* mRNA abundance than the *Cs/Cs* leaves (Fig. 3a). In normal conditions, the leaves of cucumber pretreated with SA and OE-*PAL*/OE-*PAL*, WT/OE-*PAL*, and OE-*PAL/*WT transgenic plants displayed an increase in *CsNPR1* expression, while *Cs/Cs* and *Cs/Cm* plants pretreated with 2-aminoindan-2-phosphonic acid (AIP) and AOPP as well as the RNAi-*PAL/*RNAi-*PAL,* WT/RNAi-*PAL*, and RNAi-*PAL*/WT plants had no difference in *CsNPR1* mRNA abundance, compared with H_2_O treatment and WT/WT plants, respectively (Fig. S7, see online supplementary material). Under chilling stress, SA significantly increase the CsNPR1 mRNA and protein expression levels in both *Cs/Cs* and *Cs/Cm* plants, while the treatment of AIP or AOPP down-regulated the CsNPR1 mRNA and protein expressions, compared with the H_2_O treatment, but the CsNPR1 mRNA and protein expressions in each treatment of *Cs/Cm* leaves was always significantly higher than that of *Cs/Cs* leaves (Fig. 3b and d). Moreover, the *CsNPR1* mRNA abundance in OE-*PAL*/OE-*PAL*, WT/OE-*PAL*, and OE-*PAL/*WT leaves was higher compared to WT/WT leaves, the RNAi-*PAL/*RNAi-*PAL,* WT/RNAi-*PAL*, and RNAi-*PAL*/WT leaves was lower compared to WT/WT leaves (Fig. 3c). Likewise, a similar result was observed at the protein level of CsNPR1 (Fig. 3e). As determined by the cell-free protein degradation assay, proteasome inhibitor (MG132) markedly inhibited CsNPR1 protein degradation, with SA treatment repressing and AIP and AOPP treatment promoting this process. CsNPR1 degradation was greatly slower in *Cs/Cm* leaves than in *Cs/Cs* leaves in all treatments (Fig. 3f). Compared to WT/WT plants, the OE-*PAL*/OE-*PAL*, WT/OE-*PAL*, and OE-*PAL/*WT improved the stability of CsNPR1 protein *in vitro*, while the RNAi-*PAL/*RNAi-*PAL,* WT/RNAi-*PAL*, and RNAi-*PAL*/WT accelerated the CsNPR1 degradation (Fig. 3g). These findings revealed that *CsNPR1* plays a role in grafting-induced cold tolerance and exogenous or endogenous overexpressed SA-induced CsNPR1 protein expression in *Cs/Cm* plants under cold stress.

### *CsNPR1* positively regulated grafted cucumber cold tolerance

**Figure 4:**
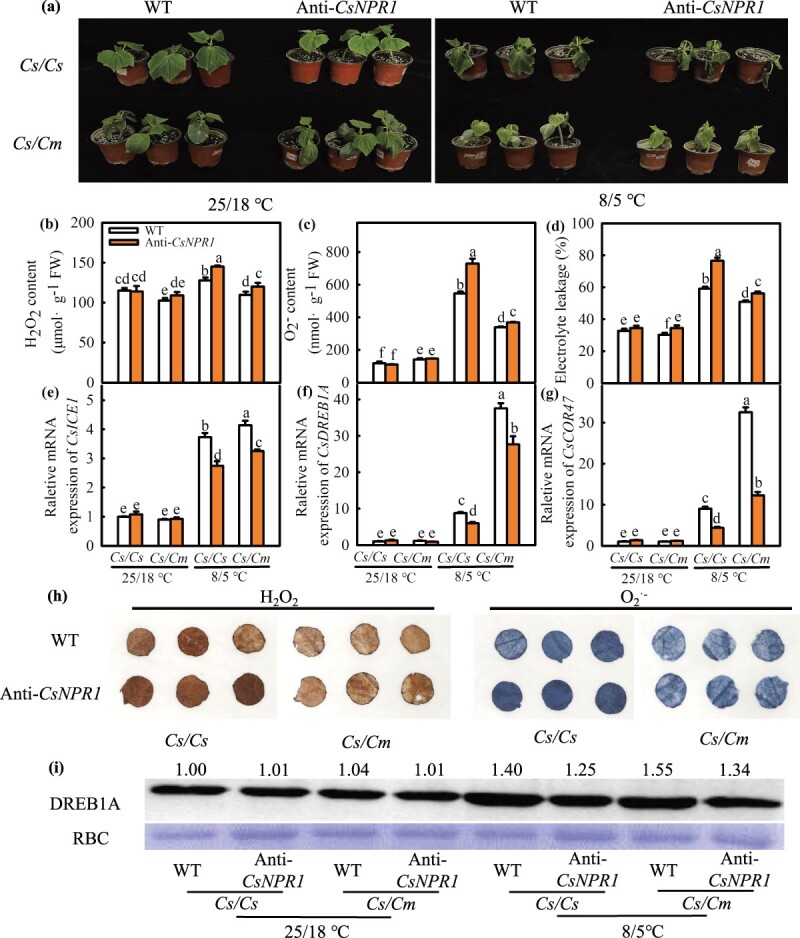
Suppression expression of *CsNPR1* reduces the cold tolerance. **(a)** Phenotype of seedlings; **(b–d)** accumulation of H_2_O_2_ and O_2_^·−^ and EL; **(e–g)** expression of *CsICE1, CsDREB1A,* and *CsCOR47*; **(h)** NBT and DAB staining of H_2_O_2_ and O_2_^·−^; **(i)** DREB1A protein. The leaves were sampled after chilling treatment at 0 h and 24 h. Data represent the mean values of four biological replicates, with standard deviations (± SDs) included. With a significance level of *P* < 0.05, different letters differ significantly between samples.

To further explore the role of *CsNPR1* response to cold stress in grafted cucumber, the Anti-*CsNPR1* transgenic plants were obtained (Fig. S8, see online supplementary material) and overexpressed *CsNPR1* transgenic leaves as scion of *Cs/Cs* and *Cs/Cm* using *Agrobacterium-*mediated genetic transformation and *Agrobacterium-*mediated transient transformation, respectively. The relative *CsNPR1* mRNA expression was significantly higher in Anti-*CsNPR1/Cm* and OE-*CsNPR1*/*Cm* leaves than in Anti-*CsNPR1/Cs* and OE-*CsNPR1*/*Cs* leaves under cold stress (Fig. S9a and b, see online supplementary material). We selected the transgenic line of Anti-*CsNPR1*–2 and analysed the effect of suppressed expression of *NPR1* (Anti-*CsNPR1*) on the cold tolerance of Anti-*CsNPR1/Cs* and Anti-*CsNPR1/Cm* plants. As shown in Fig. 4a–d, the plant phenotype, H_2_O_2_ and O_2_^·−^ accumulation or EL between Anti-*CsNPR1* and WT plants have no significant differences at optimum growth temperature (25/18°C). Under cold stress, all plants exhibited wilting and a significantly increased H_2_O_2_ and O_2_^·−^ accumulation and EL, but the Anti-*CsNPR1* plants exhibited severe water loss and considerably higher H_2_O_2_ and O_2_^·−^ accumulation and EL than the WT plants. The H_2_O_2_, O_2_^·−^ and EL in Anti-*CsNPR1* leaves were elevated by 27.1%, 562.8%, and 122.2%, respectively, and those in WT leaves were elevated by 11.1%, 361.7%, and 80.7%, respectively. The Anti-*CsNPR1/Cm* plants exhibited significantly lighter cold damage as well as the H_2_O_2_ and O_2_^·−^ accumulation and EL in Anti-*CsNPR1/Cm* plants elevated by 7.38%, 140.7%, and 68.1%, but that in Anti-*CsNPR1/Cs* plants declined by 10.1%, 151.2%, and 62.9%, respectively, under chilling stress. H_2_O_2_ and O_2_^·−^ accumulation was also analysed via DAB and NBT staining, and the results corroborated the biochemical measurement results (Fig. 4h). In addition, the expression of *CsICE1*, *CsDREB1A*, and *CsCOR47* significantly upregulated at 5°C. While the Anti-*CsNPR1* leaves displayed a strikingly lower cold-responsive genes expression than the WT leaves, the Anti-*CsNPR1/Cm* plants had markedly higher values than the Anti-*CsNPR1/Cs* plants (Fig. 4e–g). The DREB1A protein level corroborated its mRNA expression result (Fig. 4i).

**Figure 5:**
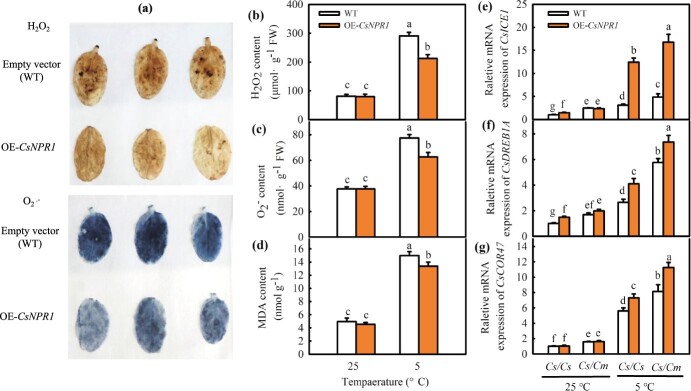
Overexpression of *CsNPR1* improves the cold tolerance of *Cs/Cs* and *Cs/Cm* cucumbers. **(a)** DAB and NBT staining of H_2_O_2_ and O_2_^·−^, respectively at 5°C; **(b–d)** Accumulations of H_2_O_2,_ O_2_^·−^ and MDA at 25°C and 5°C, respectively; **(e–g)**  *ICE1, DREB1A*, and *COR47* expression at 25°C and 5°C, respectively. Empty vector (WT)/*Cs*, *OE-CsNPR1/Cs*, and WT/*Cm*, *OE-CsNPR1*/*Cm* transient transgenic cucumbers were treated at 5°C for 12 h. Data represent the mean values of four biological replicates, with standard deviations (± SDs) included. With a significance level of *P* < 0.05, different letters differ significantly between samples.

Then, we examined the impact of overexpression of *NPR1* (OE*-CsNPR1*) on the cold tolerance of cucumber plants. The H_2_O_2_, O_2_^·−^, and MDA content in the empty vector control (WT) leaves increased by 201.9%, 258.3%, and 105.8% at 5°C, respectively, but that in OE*-CsNPR1* leaves increased by 195.7%, 166.7%, and 66.6%, respectively. OE*-CsNPR1* was found to markedly decrease ROS accumulation and MDA content in comparison to the WT (Fig. 5a–d). Moreover, the mRNA abundances of cold-responsive genes in the OE*-CsNPR1/Cs* and OE*-CsNPR1/Cm* were detected. *CsICE1, CsDREB1A*, and *CsCOR47* expression levels in OE*-CsNPR1/Cm* leaves were upregulated by 16.8-, 7.4-, and 11.3-fold, respectively, and in OE*-CsNPR1/Cs* leaves were upregulated by 12.4-, 4.1-, and 7.3-fold, respectively, following at 5°C for 12 h (Fig. 5e–g). Overall, these findings demonstrate that *CsNPR1*, as a positive regulator of cold response, participates in grafting-induced cold tolerance.

### *CsICE1* overexpression improved the cold stress response of grafted cucumbers

**Figure 6:**
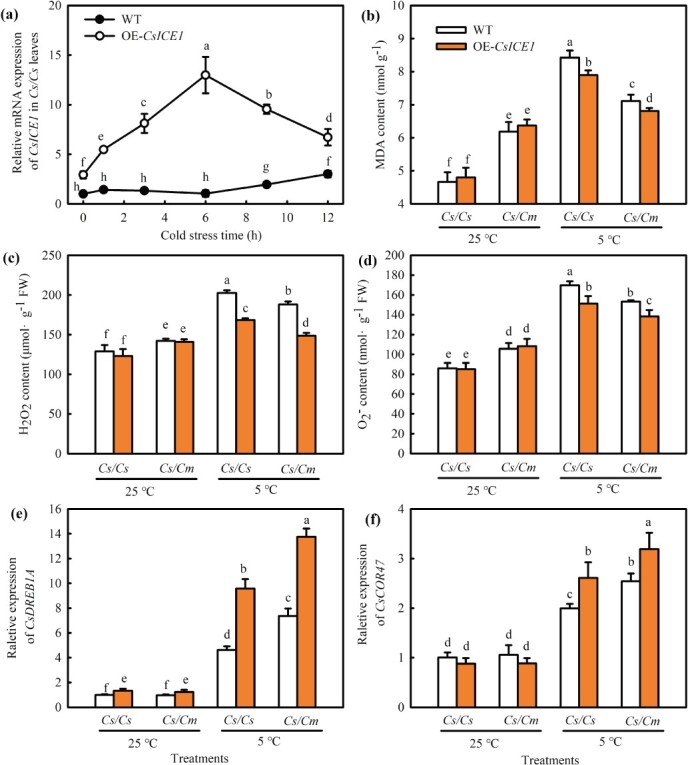
Overexpression of *CsICE1* improves the cold tolerance of *Cs/Cs* and *Cs/Cm* cucumbers. **(a)**  *CsICE1* expression in grafted cucumbers under cold stress. **(b–d)** MDA content, H_2_O_2_ and O_2_^·−^ accumulation at 25 and 5°C, respectively; **(e, f)**  *DREB1A* and *COR47* expression at 25 and 5°C, respectively. Empty vector (WT)/*Cs*, *OE-CsICE1/Cs*, and WT/*Cm*, *OE-CsICE1*/*Cm* transient transgenic cucumber plants were treated at 5°C for 12 h. Data represent the mean values of four biological replicates, with standard deviations (± SDs) included. With a significance level of *P* < 0.05, different letters differ significantly between samples.

ICE, a key TF of the cold signaling pathway, is crucial for regulating plant response to cold signals. Among the upregulated genes, an ICE gene named *CsICE1* (CsaV3_3G027730) was induced by *Cm* (Figs S1e and S5b, f, see online supplementary material). Because SA treatment and OE*-CsNPR1* upregulated *CsICE1* expression (Fig. 5e; Fig. S2d, see online supplementary material) in *Cs/Cm*, we then investigated whether *CsICE1* participated in the cold tolerance of grafted cucumber. The results reveled that cold stress improved the *CsICE1* mRNA level in *Cs/Cs* seedlings, with pronounced improvement at 6-h treatment (Fig. 6a). In order to further investigate the role of *CsICE1* in the cold stress response in grafted cucumber, the *Cs/Cs* and *Cs/Cm* plants of OE-*CsICE1* transient transgenic leaves as scion were obtained (Fig. S9c, see online supplementary material). After subjecting to 5°C for 12 h, the H_2_O_2_, O_2_^·−^ and MDA content in the WT leaves increased by 57.1%, 97.3%, and 80.6%, respectively, but that in OE*-CsICE1* leaves only increased by 36.9%, 77.8%, and 64.5%, respectively (Fig. 6b–d). Compared with WT, the mRNA abundance of *CsDREB1A* and *CsCOR47* in OE-*CsICE1/Cm* leaves was upregulated by 11.2- and 3.60-fold, but that in OE-*CsICE1/Cs* leaves only increased by 7.58- and 2.41-fold, respectively, under chilling stress (Fig. 6e and f). Overall, these findings indicate that *CsICE1* positively modulates the cold tolerance of grafted cucumber.

### CsNPR1 directly interacted with CsICE1

**Figure 7:**
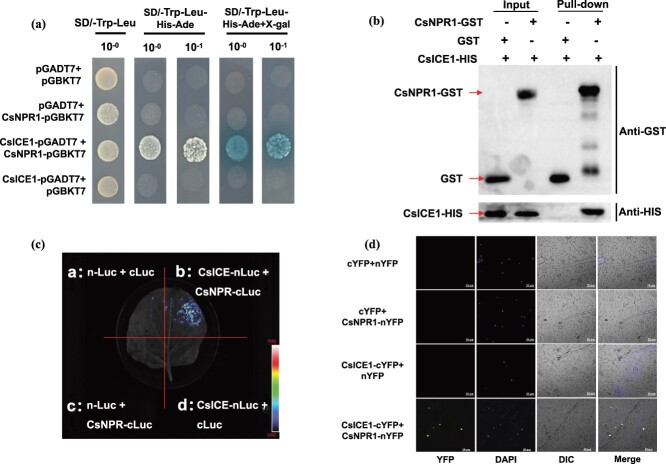
CsNPR1 directly interacts with CsICE1. **(a)** The Y2H experiment is used to determine the interactions between *CsNPR1* and *CsICE1*., the co-transformation yeast cells were plated on SD/−Trp-Leu (SD/−T-L) and SD/−Trp-Leu-His-Ade (SD/−T-L-H-A) media with 150 ng·ml^−1^ AbA for 2 d, and 10^−0^ and 10^−1^ represent the dilutions of yeast solution. The positive interactions were further confirmed using X-α-gal. **(b)** Pull-down assay to identify the *in-vitro* interaction of CsNPR1 and CsICE1. The CsICE1-HIS proteins were respectively incubated with CsNPR1-GST or GST proteins, and then pulled down by GST purification kit. Immunoblotting using anti-GST and anti-His antibodies allowed for the detection of the eluted proteins. **(c)** Luciferase complementarity assay to identify the *in-vivo* interactions of CsNPR1 with CsICE1 in tobacco. **(d)** BiFC assay showing the *in-vivo* interactions of CsNPR1 with CsICE1 in tobacco. Bars = 50 μm. Similar results were seen in all three of the experiments.

Considering that *CsNPR1* and *CsICE1* exhibit similar expression patterns under cold stress and that ICE1 is crucial for plants to respond to cold signal, we hypothesize that CsNPR1 may interact with CsICE1 to contribute to grafting-induced cold tolerance. An *in vitro* detection experiment was conducted based on yeast two-hybrid (Y2H) analysis. To avoid CsNPR1 auto-toxication, we deleted the 3′ region of CsNPR1 (561aa–585aa) to construct the *CsNPR1*-pGBKT7* vector (the trimmed *CsNPR1* sequence is shown in Table S1, see online supplementary material) and used 150 ng/ml Aureobasidin A (AbA) to inhibit *CsNPR1*–pGBKT7* auto-activation (Fig. S10, see online supplementary material). As determined by the Y2H assay results, only the co-transformed combination with CsICE1 and CsNPR1 yeast cells grew on SD (−Trp/−Leu/-His/−Ade/+X-α-gal) media, whereas other combinations all failed to induce growth on the same media (Fig. 7a), indicating that CsNPR1 can directly interact with CsICE1. To verify this result, *in vitro* pull-down assay was performed. As shown in Fig. 7b, the CsNPR1-GST fusion protein was bound by CsICE1-HIS, while the GST control did not bind. *In vivo* dual-luciferase assays conducted to validate CsNPR1–CsICE1 interaction revealed a fluorescence signal in the co-transformation region of CsNPR1 and CsICE1 (Fig. 7c). The interaction between CsNPR1 and CsICE1 was further confirmed using a bimolecular fluorescence complementation (BiFC) assay (Fig. 7d). These findings establish that CsNPR1 can directly interact with CsICE1 *in vitro* and *in vivo* to modulate the cold stress response in grafted cucumber.

### CsNPR1–CsICE1 interaction promoted the rootstock-induced cold tolerance of cucumber

**Figure 8:**
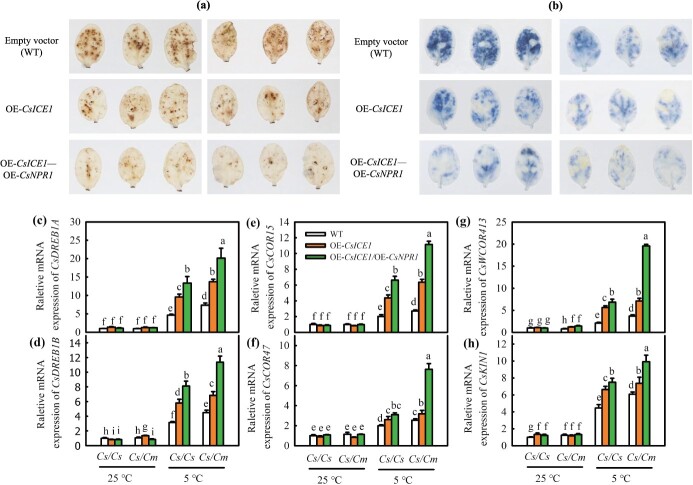
Co-overexpression of *CsNPR1* and *CsICE1* improves the cold tolerance of *Cs/Cs* and *Cs/Cm* cucumbers. **(a, b)** DAB and NBT staining of H_2_O_2_ and O_2_^·−^, respectively; **(c–h)** the expression of *DREB1A, DREB1B*, *COR15, COR47, COR413*, and *KIN1* of empty vector (WT), *OE-CsICE1* and *OE-CsICE1-CsNPR1* transient transgenic cucumber leaves, respectively, at 25 and 5°C for 9 h. Data represent the mean values of four biological replicates, with standard deviations (± SDs) included. With a significance level of *P* < 0.05, different letters differ significantly between samples.

To verify the role of the CsNPR1–CsICE1 interaction in regulating cold tolerance, we obtained the *Cs/Cs* and *Cs/Cm* plants of *CsNPR1* and *CsICE1* co-expressed (OE*-CsNPR1*–OE*-CsICE1*) transient transgenic leaves as scion (Fig. S11, see online supplementary material). OE-*CsNPR1*–OE-*CsICE1* significantly decreased the accumulation of H_2_O_2_ and O_2_^.−^ in detached leaves induced by cold stress, as determined by NBT and DAB staining, in comparison to OE-*CsICE1*. The decrease in H_2_O_2_ and O_2_^.−^ accumulation in OE*-CsNPR1*–OE*-CsICE1/Cm* was markedly greater than that in OE*-CsNPR1*–OE*-CsICE1/Cs* under cold stress (Fig. 8a and b). OE*-CsNPR1*–OE*-CsICE1* co-expressed leaves showed much higher mRNA abundances of the key downstream genes of *ICE1*, including *CsDREB1A, CsDREB1B, CsCOR47, CsCOR15, CsWCOR413,* and *CsKIN1* than OE*-CsICE1*. Compared with OE*-CsNPR1*–OE*-CsICE1/Cs* leaves, a greater improvement in the *ICE1* downstream gene mRNA level was observed in OE*-CsNPR1*–OE*-CsICE1/Cm* leaves at 5°C (Fig. 8c–h). Overall, these results suggest that the CsNPR1–CsICE1 interaction positively regulates the rootstock-induced cold tolerance of cucumber.

## Discussion

Plants’, especially solanaceous fruit and melon vegetables, response to abiotic stresses including heat, cold, and salinity can be enhanced by grafting with resistant rootstocks. In this study, grafted seedlings showed lower CI, EL, and MDA content, as well as higher expression of chilling response genes, such as *DREB1A* and *ICE1* (Fig. S1, see online supplementary material), corroborating previous findings [7, 34]. Despite the importance of long-distance signaling molecules like phytohormones, water and mineral ion transport, osmotic substances, miRNAs, and proteins in rootstock–scion interaction, how these signaling molecules, especially phytohormones signals, participate in regulating abiotic stress resistance in grafting plants remains unclarified.

SA is a crucial phytohormone that plays a significant role in the plant resistance to chilling stress [28]. Here, we also discovered that SA could alleviate damage to the cucumber photosystem and stimulate the expression of cold responsive genes (*ICE1, DREB1A, COR47*) in both *Cs/Cs* and *Cs/Cm* plants when exposed to cold stress (Fig. S2, see online supplementary material). The application of SA synthesis inhibitors AIP and AOPP dramatically reduced the chilling tolerance of cucumbers enhanced by grafting, highlighting SA involvement in grafting-induced cold tolerance. Although SA, as a long-distance transduction signaling molecule, could participate in rootstock–scion information exchange [44, 45, 46], the molecular verification of SA function during this process was limited. Previous research has informed that the SA synthesis is approximately 90% dependent on the ICS pathway in Arabidopsis, with the PAL pathway producing the isomer of SA, 4-hydroxybenzoic acid, instead of SA [46]. However, different SA synthesis pathways differ greatly in different species. Under low temperature, the SA content and the *AtICS* expression had no significant changes in Arabidopsis for 7 days [47, 71], whereas the SA content was significantly accumulated at 1.4- and 2.2-fold in cucumber and watermelon within 1 day, respectively [27, 48]. The PAL pathway has been evidenced as the main pathway for SA synthesis in cucumbers responding to chilling stress [27, 28, 34]. Thus, we obtained *CsPAL* transgenic cucumber plants and found that *CsPAL* overexpression markedly increased SA content, whereas *CsPAL* inhibition declined SA content (Fig. S3, see online supplementary material). Notably, OE*-PAL*/OE*-PAL* with *CsPAL* overexpression not only alleviated chilling damage of ungrafting seedlings, as indicated by lower EL, MDA content, ROS accumulation, CI, and relative mRNA expression of *CsICE1*, *CsDREB1A*, *CsDREB1B*, and *CsCOR47* in comparison to WT, but also promoted the cold tolerance of WT/OE-*PAL* and OE-*PAL*/WT grafting seedlings*.* RNAi*-PAL* with *CsPAL* inhibition showed the opposite results (Fig. 1). To further explore SA participation in rootstock–scion interaction, we measured the change in *CsPAL* mRNA abundance, PAL activity, and SA content in both the leaves and roots of grafted and ungrafted transgenic plants. It was demonstrated that at both room temperature and low temperature, the OE-*CsPAL* plants shown significantly higher *CsPAL* mRNA abundance, PAL activity, and SA content in the leaves and roots. In accordance with this, these three indices for WT/OE-*PAL* in both leaves and roots exceeded those for the WT/WT leaves and roots, respectively, after 12-h chilling stress. However, RNAi-*PAL* decreased PAL activity and SA content in both leaves and roots of WT/*RNAi-PAL*, compared with WT/WT seedlings. It means the SA decline in rootstock caused by *CsPAL* inhibition is the main cause for the weakening of chilling tolerance in WT/RNAi-*CsPAL* seedlings. Notably, the SA content of xylem sap in WT/OE-*PAL* was significantly higher than that in WT/WT plants and WT/RNAi-*PAL* was lower than WT/WT plants (Fig. 2). Furthermore, these three indices of roots in RNAi-PAL/WT and OE-PAL/WT had no difference, demonstrating that the SA dependence on the PAL pathway in rootstock-scion communication played the vital role in grafting-induced chilling tolerance of cucumber.

SA signal transduction is primarily initiated by the specific binding between SA and its receptor NPR1, after which NPR1 interacts with TGA, WRKY, NIMIN, or other TFs to activate the downstream genes’ expression during plant immunity [49]. Transcriptome analysis showed significant difference in SA signal transduction-related genes expression between *Cs/Cs* and *Cs/Cm* plants at low temperatures. This indicates that the SA signaling pathway takes part in the cold tolerance of cucumber induced by grafting (Fig. S5, see online supplementary material). Considering the significance of NPR1 in the SA signaling pathway, the change in *CsNPR1* attracted our attention. Chilling stress was previously shown to upregulate *AtNPR1* expression in Arabidopsis [50]. In our study, the data of real-time PCR verification and further KEl pathway analysis of the SA signal transduction-related DGEs both proved the upregulation of *CsNPR1* in *Cs/Cm* plants, and the nucleotide sequences of *CsNPR1* were highly conserved (Figs S5 and 6, see online supplementary material). Moreover, exogenous and endogenous over-expressed SA increased *CsNPR1* mRNA and protein expression and reduced CsNPR-HIS degradation in both *Cs/Cs* and *Cs/Cm* plants, whereas endogenous SA inhibition blocked this induction effect. However, pumpkin-grafted plants exhibited a higher *CsNPR1* level following AIP and AOPP treatments than the *Cs/Cs* plants under cold stress (Fig. 3), indicating that *CsNPR1* expression was mediated by the raising of SA content in scion or grafting under low temperature stress. Cao et al. [71] and Glazebrook *et al.* [51] revealed that *npr1* displayed increased susceptibility to pathogens and decreased pathogenesis-related (PR) gene expression; however, NPR1 overexpression in *npr1* could restore the resistance of disease and PR expression in Arabidopsis [72]. The same results were obtained under cold stress. As an example, the cold resistance of *npr1* is much weaker than that of WT, and OE*-AtNPR1* enhances the cold adaptation of Arabidopsis [37]. In our study, Anti-*CsNPR1* as the scion notably reduced the cold tolerance of *Cs/Cm* seedlings, as proved by the higher ROS content and EL and the lower mRNA abundance of *CsICE1*, *CsDREB1A*, and *CsCOR47* (Fig. 4), whereas OE*-CsNPR1* improved cold tolerance with decreased MDA and ROS accumulation and upregulated cold responsive genes expression in comparison to the WT seedlings (Fig. 5). However, the cold tolerance of cucumber induced by grafting is a very complex process, there are many other signals involved in this process, such as SA, ABA, MeJA, MT, H_2_O_2_, mRNA, etc. [3, 7, 52, 53]. These signals not only have function singly, but also interact with each other to regulate graft-induced cold tolerance in cucumber and thus the cold tolerance of Anti-*CsNPR1*/*Cm* was still better than that of Anti-*CsNPR1*/*Cs* under chilling stress. According to the above results, we speculated that SA-induced *CsNPR1* expression could positively participate in the regulation of cold tolerance in grafted cucumber.

Due to NPR1 lacking the DNA-binding domain and only acting as a coactivator [54, 55], it needs to interact with other proteins. For example, NPR1 mediated the WRKY18–WRKY60 module to activate *ABI4* and *ABI5* expression and then positively regulated the ABA signal [56]. Meanwhile, NPR1 induced plant chilling tolerance by interacting with HSFA1 [37]. ICE, a bHLH-type TF, is believed to be a CBF inducer. Under chilling stress, ICE could bind with a *CBF* promoter, hence upregulating the downstream genes expression level that respond to cold, such as *COR15A, KIN1*, *COR47,* and *RD29A*, and promoting the cold tolerance of plants [57–59]. Here, we found that *CsICE1* overexpression declined MDA and ROS accumulation and improved *CsDREB1A* and *CsCOR47* expression under cold stress, and OE-*CsICE1/Cm* plants showed lower MDA and ROS accumulation and higher *CsDREB1A* and *CsCOR47* expression than OE-*CsICE1/Cs* plants (Fig. 6). TF complexes often regulate cold signals more effectively than single TF, and ICE–CBF can be regulated by multifarious TFs or proteins and then take part in the hormone signaling pathway to regulate chilling stress [21, 22]. For instance, SnRK2.6/OST1, which participate in the ABA signal pathway, interacts with ICE1 to yield phosphorylated ICE1, which then activates *CBF–COR* gene expression, thereby conferring low-temperature resistance to this gene [18]. Because *CsICE1* and *CsNPR1* exhibited comparable variations under chilling stress (Figs 3a and 6a; Fig. S5, see online supplementary material), we speculated whether the CsICE1–CsNPR1 interaction existed under chilling stress. Fortunately, Y2H, pull-down, luciferase complementation and BiFC experiments proved that CsNPR1 actually interacted with CsICE1 (Fig. 7), and the co-transformation of *CsNPR1* and *CsICE1* to cucumbers further demonstrated that the CsNPR1–CsICE1 module participated in regulating cold tolerance in grafted cucumber seedlings. Compared to WT, grafting seedlings with single *CsICE1* overexpression showed lower MDA and ROS accumulation and higher mRNA expression of *CsDREB1A, CsDREB1B, CsCOR47, CsCOR15a, CsWCOR413*, and *CsKIN1*. Notably, co-transforming CsNPR1 with CsICE1 notably alleviated ROS accumulation and enhanced the downstream chilling-sensitive genes expression (Fig. 8).

## Conclusion

**Figure 9:**
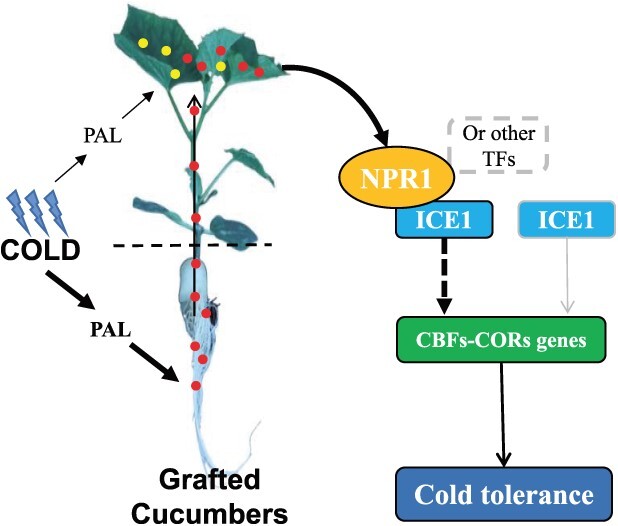
A possible model of SA involved in rootstock-scion communication to improving the cold tolerance of grafted cucumber. The yellow solid circle means SA synthesized from leaf and the red solid circle means SA synthesized from root.

In conclusion, SA was involved in regulating the cold tolerance of grafted cucumber which is induced by using pumpkin as the rootstock. The SA content increase in *Cs/Cm* seedling leaves, which was largely dependent on the PAL pathway, was not only synthesized in leaves but also transported upward from the rootstock under chilling stress. Cold stress stimulated the *CsNPR1* and *CsICE1* expression and enhanced cold tolerance in grafted cucumber. More importantly, the SA signal in scion also upregulated the *CsNPR1*–*CsICE1* module to positively mitigate lipid peroxidation damage and promote the cold responsive genes expression, resulting in improving the cold tolerance of grafted cucumber (Fig. 9).

## Materials and methods

### Plant materials and growth conditions

Cucumber (*C. sativus* L., *cv.* ‘Jinyou 35’, *Cs*), pumpkin (*Cucurbita moschata* D., *cv.* ‘Jinmama 519’, *Cm*), phenylalanine ammonia-lyase (PAL) gene overexpression, and RNAi-silenced transgenic cucumber lines were used as the experimental materials. The transgenic plant lines were obtained using the *Agrobacterium tumefaciens* genetic transformation system, and the cucumber cultivar ‘Xintaimici’ was utilized as the wild type (WT). The grafting procedure was conducted using the top insertion method once the cotyledons of both the rootstock and scion seedlings had fully expanded. The resulting self-root and hetero-root grafted seedlings, as well as the transgenic plants, were cultivated in an artificial climate chamber with the following conditions: temperature of 26/18°C (day/night), photon flux density (PFD) of 600 μmol m^−2^ s^−1^, photoperiod of 11/13 h (light /dark), and relative humidity of 80%.

### Chilling and SA treatments

All experiments were conducted in an artificial climate chamber to investigate the impact of grafting on cucumber cold tolerance. Two types of cucumber plants were used: the self-rooted (cucumber as rootstock and scion, *Cs/Cs*) and grafted cucumber plants (with pumpkin as rootstock, *Cs/Cm*). These plants were treated at 8°C/5°C for 24, 72, and 120 h, with those treated at 25°C/18°C serving as controls. The chilling injury index (CI), reactive oxygen (ROS) accumulation, and cold-responsive genes expression were measured at 72 h.

To determine the role of SA in grafting-induced cold tolerance, *Cs/Cs* and *Cs/Cm* plants were sprayed with SA (1.0 mM), SA synthesis inhibitors 2-aminoindan-2-phosphonic acid (AIP, 0.03 mM), L-2-aminooxy-3-phenylpropionic acid (AOPP, 0.1 mM), or distilled water (control) for 24 h and then the plants were treated at 5°C for 48 h to determine the photosynthetic rate (Pn) and *RCA, rbcL*, and cold-responsive genes expression.

Subsequently, WT/WT, overexpressed *CsPAL* (OE-*PAL*/OE-*PAL*), and RNAi (RNAi-*PAL*/RNAi-*PAL*) self-grafted transgenic plants and WT/*OE-PAL,* WT/RNAi-*PAL*, and *OE-PAL*/WT*,* RNAi-*PAL* /WT grafted plants were exposed to cold conditions for 12 or 72 h in order to assess the electrolyte leakage (EL), malondialdehyde (MDA) content, hydrogen peroxide (H_2_O_2_) and superoxide anion (O_2_^.−^) contents, cold-responsive genes expression, as well as SA content, PAL activity, and *CsPAL* expression.

At the two-leaf stage, the transgenic plants of Anti-*CsNPR1/Cs* and Anti-*CsNPR1*/*Cm* were subjected to 8°C/5°C for 24 h, with those plants subjected to 25°C/18°C serving as controls. The EL, MDA content, ROS accumulation, and the cold-responsive genes expression were determined. Meanwhile, the pBI121-GFP empty vectors, as well as overexpressed *CsNPR1* and *CsICE1* vectors were injected into the cotyledon leaves of *Cs/Cs* and *Cs/Cm* seedlings. After a 12-h dark incubation, half of the WT and overexpressed leaves of *CsNPR1* and *CsICE1* were treated at 5°C and the other half were treated at 25°C. The cold-responsive genes expression, MDA content, and ROS accumulation were determined after chilling for 9 or 12 h.

### Measurement of CI, EL, MDA, and ROS accumulation

The CI was estimated using the method in [60]. The calculation formula is as follows: CI = Σ (plants of different grade × grade)/[total plants × 5 (the maximum grade)]. Grades in the formula represent the cold damage degree of plants. The EL was determined using the approach [61]. The measurement of electrical conductivity (EC) was conducted with a DDB-303A conductivity meter (Lei—ci, Shanghai, China). The initial EC and final EC (A/B) were used to calculate EL, where A is the EC detected after incubating leaves in distilled water at 25°C for 3 h, and B is the EC detected after boiling for 30 min and cooling to room temperature. The MDA content was determined following the described method by Zhao and Cang [62] using the thiobarbituric acid (TBA) colorimetric approach. For H_2_O_2_ determination, the H_2_O_2_ kit (Njjcbio, Nanjing, China) was utilized by the guideline description provided. The levels of O_2_^.−^ content were assessed following the method outlined by Li and Gong [63]. Qualitative analysis of H_2_O_2_ and O_2_^.−^ were conducted using a 3, 3-diaminobenzidine (DAB) kit (Solaribio, Beijing, China) and nitroblue tetrazolium (NBT) (Biotopped, Beijing, China), as outlined in the studies by Thordal-Christensen *et al.* [64] and Jabs *et al.* [65].

### SA content and PAL activity assay

SA content quantification was carried out following the previously described method [34]. In brief, freeze-dried cucumber leaves and roots were extracted with 80% methanol for 16 h, and the resulting supernatants were separated by centrifugation and then evaporated to dryness using a rotary evaporator (EYELA N-1210B, Shanghai, China). Following the elimination of pigments and phenolic impurities using chloroform and polyvinylpolypyrrolidone (PVPP), SA was extracted into ethyl acetate, rotary evaporated until dry, and then dissolved in 50% methanol. The xylem sap was collected beneath the scion cotyledon, and the EP tube was filled with absorbent cotton and closely contacted with the section on the stem. After absorption for 10 h, the absorbent cotton was weighed and leached in 80% methanol for 16 h, then concentrated and extracted with 50% methanol. The extract was filtered via a 0.22 μm filter and analysed using TSQ Quantum Access (Thermo Fisher Scientific, Waltham, MA, USA) for measurement. The PAL activity measurement followed the protocol described by Shang *et al.* [29].

### Transcriptome analysis

*Cs/Cs* and *Cs/Cm* plants were subjected to cold stress for 12 h, while plants grown at 25°C served as controls. We extracted total RNA from leaves of *Cs/Cs* and *Cs/Cm* plants, both before and after chilling stress, and performed transcriptome analyses using the method described by Fu *et al.* [34]. Each sample was replicated three times. To determine the differential genes, we applied a significant threshold of *P-*value <0.05 and fold change >1.5. The data of this study have been deposited in the Gene Expression Omnibus of NCBI and can be accessed through SRA accession: PRJNA701131230 (http://www.ncbi.nlm.nih.go v/bioproject/701131).

### Vector construction and genetic transformation

The coding sequence (CDS) of *CsPAL*, *CsNPR1*, and *CsICE1* was inserted into the pBI121-GFP vector, which contains the CaMV35S promoter, to create overexpression vectors. The pBI121-GFP vector was used to insert a 432-bp fragment sequence of *CsNPR1* (Table S2, see online supplementary material) to generate antisense vectors. Similarly, the pBWA(V)KS vector was used to insert a 201-bp fragment sequence of *CsPAL* (Table S3, see online supplementary material) to generate RNAi-generated vectors. Table S4 (see online supplementary material) provides a comprehensive list of the primers utilized in this work.

Using the freeze–thaw procedure, the recombinant vector plasmids were transformed into Agrobacterium LBA4404 (Weidi, Shanghai, China). Peeling cotyledons were used as explants for the Agrobacterium-mediated genetic transformation of cucumbers [66]. To perform transient transformation of cucumber leaves, we selected cucumber leaves that exhibited consistent growth and then injected *Agrobacterium* containing overexpression vectors of *CsPAL*, *CsNPR1*, or *CsICE1* into the cucumber leaves using a medical syringe. The transgenic plants were detected by PCR analysis.

### RNA extraction and qRT–PCR analysis

Using an RNA extraction kit (Trizol, TRANs, Beijing, China), total RNA was extracted from plant materials and reverse transcription to cDNA using HiScript® III RT SuperMix (Vazyme, Nanjing, China). The mRNA abundance of cold responsive genes, rubisco large-subunit, transcriptome-related genes, and *CsNPR1* in cucumber leaves were assessed by quantitative real-time PCR (qRT–PCR) using the LightCycler® 480II equipment (Roche, Penzberg, Germany). β-actin served as the control. Table S5 (see online supplementary material) includes the primers designed for the genes.

### Protein degradation and western blot analysis

A protein degradation buffer containing 25 mM Tris–HCl (pH = 7.5), 10 mM MgCl_2_, 10 mM NaCl, 10 mM ATP, 5 mM dithiothreitol (DTT), and 4 mM phenylmethanesulfonyl fluoride (PMSF) was used to extract the total protein from the cucumber leaves. Equal amounts of extracted proteins and *Escherichia coli* BL21(DE3) (Vazyme, Nanjing, China) induced CsNPR1-HIS recombinant proteins were subjected to incubation at 22°C for the specified sampling times. For protease inhibitor MG132 treatment (as control), cucumber leaf extracts were pretreated with DMSO (dissolve agent of MG132) or 50 μM MG132 for 30 min and then mixed with the CsNPR1–MG132 protein and incubated at the specified sampling times. The relative protein level of CsNPR1 was detected by western blotting (Tanon, Shanghai, China) with anti-His-tag mouse monoclonal antibody (CWBio, Beijing, China). Western blotting was conducted as previously mentioned [34].

### Yeast two-hybrid (Y2H) assay

The CDS of *CsNPR1* (remove self-toxic sequence) was cloned into the pGBKT7 bait vector, the *CsICE1* CDS was cloned into the pGADT7 prey vector. The recombinant plasmid combinations were co-transferred into Y2H-sensitive state cells. The transformed yeast strains were grown on SD/−Trp/−Leu medium, and the resulting colonies were transferred to SD/−Trp/−Leu/-His/−Ade medium for the screening of interacting proteins. Table S4 (see online supplementary material) contains the primers used in this experiment.

### Pull-down assay

The insertion of *CsNPR1* CDS into the pGEX-4 T-1 vector yielded the glutathione S-transferase (GST)-tag fusion protein, whereas the insertion of *CsICE1* CDS into pET-32a (+) yielded the HIS-tag fusion protein. The fusion expression proteins of GST-*CsNPR1* and HIS-*CsICE1* were produced by 1 M isopropyl-β-d-thiogalactoside (IPTG) induction after these recombinant plasmids were transformed into the *E. coli* strain BL21 (DE3). The pull-down assays were conducted with the BeaverBeads™ GSH (Suzhou, China) according to the instructions. Table S4 (see online supplementary material) contains the primers used in this experiment.

### Luciferase complementation imaging assays

*CsNPR1* and *CsICE1* coding regions were cloned into the pCAMBIA1300-cLUC and pCAMBIA1300-nLUC vectors, respectively. Tobacco leaves were injected with GV3101(pSoup) *Agrobacterium* strain solutions carrying recombinant plasmids using syringes. An imaging system (Tanon, Shanghai, China) was used to create the image. The primers used in this experiment are listed in Table S4 (see online supplementary material).

### Bimolecular fluorescence complementation assays

The CDS of *CsNPR1* and *CsICE1* were inserted into pSm35s-nYFP-ccdb and PpSm35s-ccdb-cYFP vectors, respectively. The solutions of *Agrobacterium* strain containing recombinant plasmids were mixed and transmitted into tobacco leaves using syringes. The YFP fluorescence was detected by a confocal laser-scanning microscope (Zeiss LSM 510 Meta, Jena, Germany). Table S4 (see online supplementary material) includes the specific primers used in this experiment.

### Statistical analysis

The data were displayed as means ± standard deviation (SD), and the complete experiment was carried out at least four times. DPS software was used to statistically evaluate all of the data. Duncan’s multiple range tests were used to evaluate the statistical analysis of the data at *P* < 0.05.

## Supplementary Material

Web_Material_uhae231
