# Supplementary material for: Salicylic acid improves chilling tolerance via CsNPR1–CsICE1 interaction in grafted cucumbers
Source: Hortic Res. 2024 Aug 9;11(10):uhae231. doi: 10.1093/hr/uhae231 (PMC11492142; doi:10.1093/hr/uhae231)
Supplement: Web_Material_uhae231 [file web_material_uhae231.zip › Supplementary File.pdf]

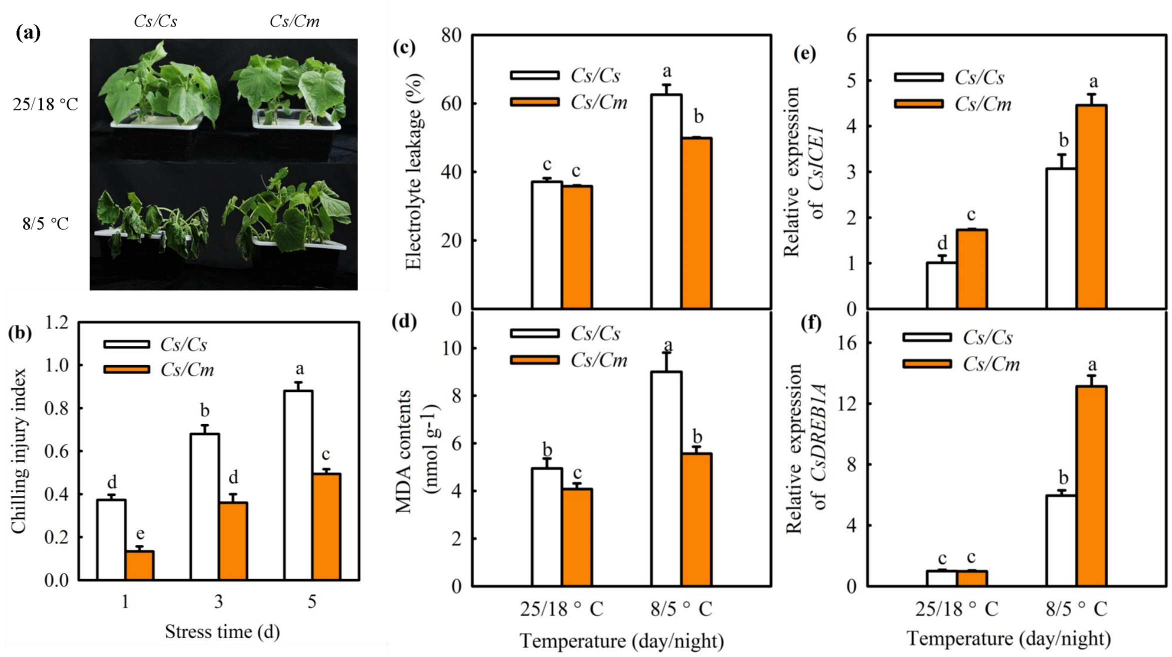

Fig. S1 Effect of rootstock on the cold tolerance of self-root and hetero-root grafted cucumbers. **(a),** Phenotype of seedlings before and after cold treatment for 72 h; **(b),** CI after cold treatment for 24-120 d; **(c-f),** EL, MDA content, and Relative mRNA expression of *ICE1* and *DREB1A*, respectively before and after cold treatment for 72 h. Data were presented as the mean of four biological replicates (± SDs). Different letters indicate a significant difference between samples at $p < 0.05$.

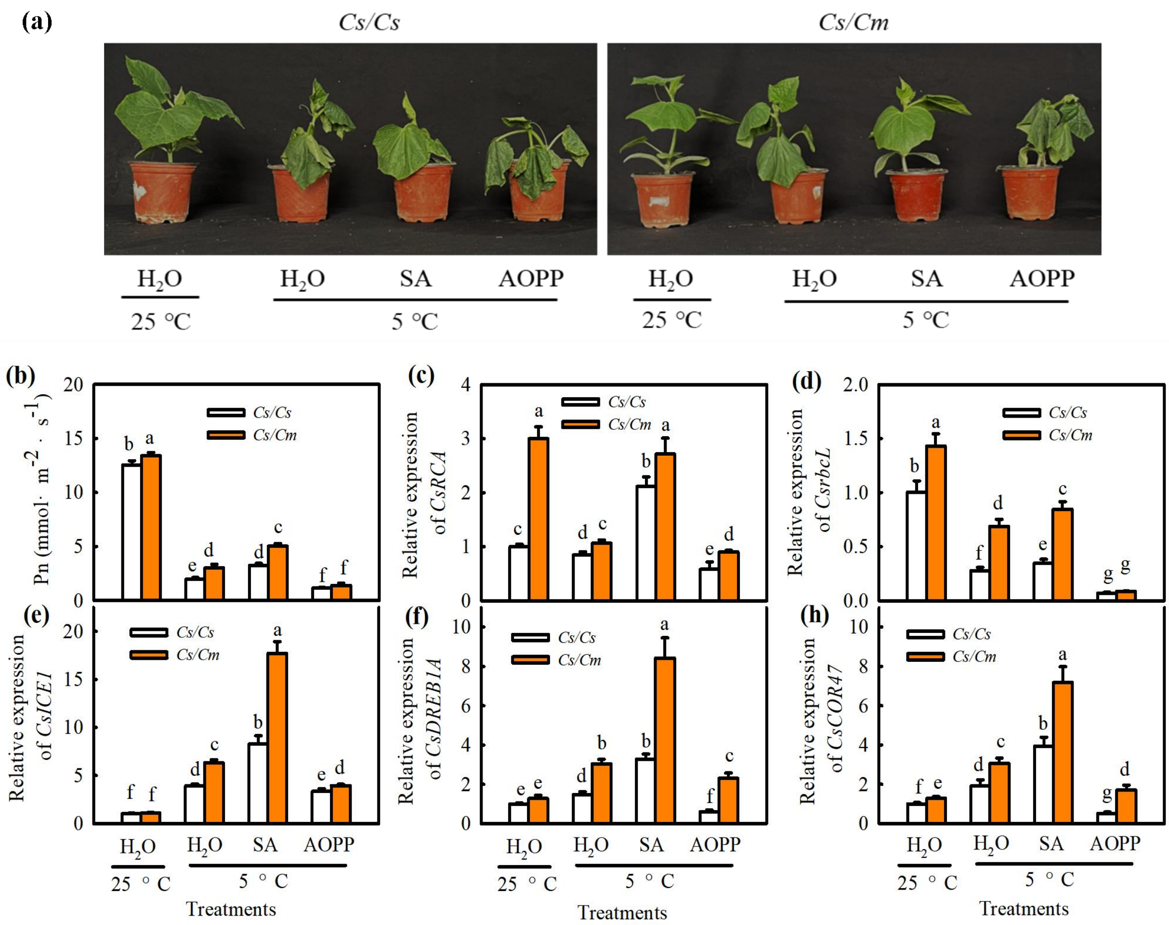

Fig. S2 SA enhances the cold tolerance of self-root and hetero-root grafted cucumbers. **(a),** Phenotype of seedlings; **(b),** Pn; **(c, d),** Relative mRNA expression of *CsRCA and CsrbcL*, respectively; **(e-g),** Relative mRNA expression of *CsICE1, CsDREB1A, and CsCOR47*, respectively. Plants with three leaves were pretreated with 1.0 mM SA, 0.1 mM AOPP, or deionized water (H_2O, control). After 24 h, the plants were exposed to 5 °C for 48 h. Data were presented as the mean of four biological replicates (± SDs). Different letters indicate a significant difference between samples at $p < 0.05$.

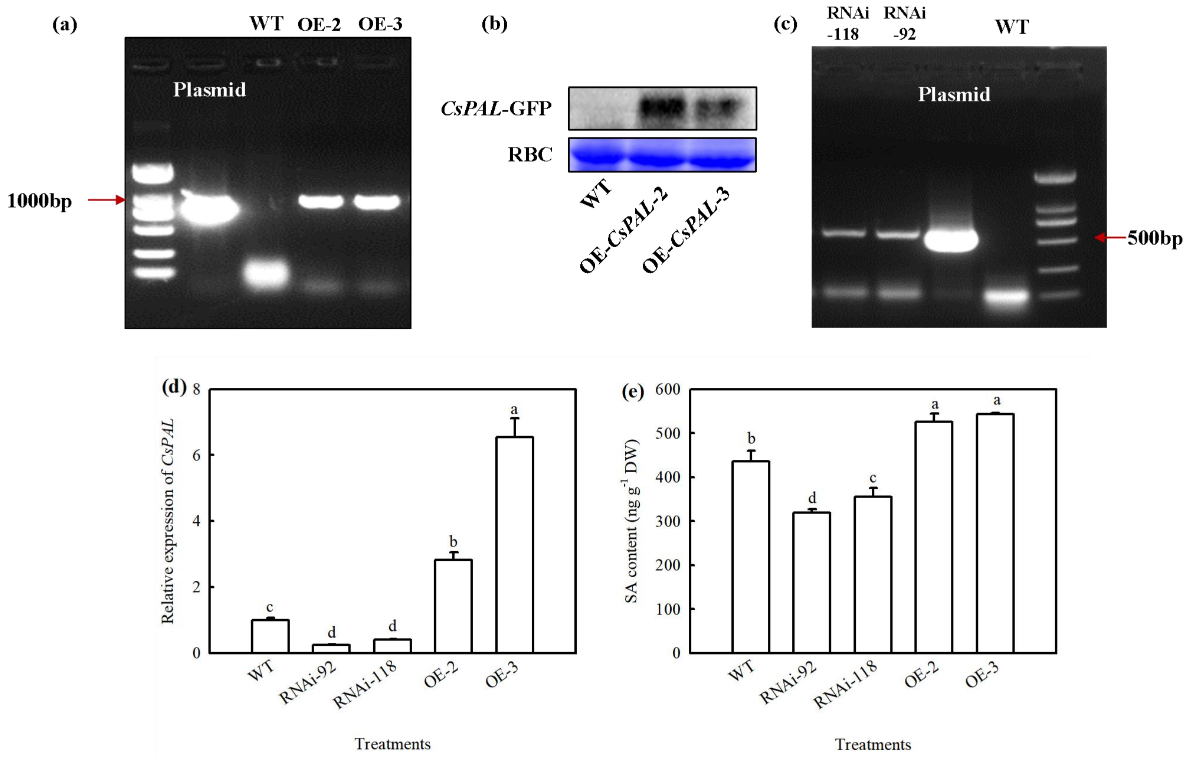

Fig. S3 Identification of OE-*CsPAL* and RNAi-*CsPAL* transgenic plants. **(a),** PCR electrophoresis results of OE-*CsPAL* plants. **(b),** Identification of GFP fusion protein in OE-*CsPAL* transgenic plants; **(c),** PCR electrophoresis results of RNAi-*CsPAL* plants **(d),** Relative mRNA expression of *CsPAL*. **(e),** SA content. Data were presented as the mean of four biological replicates (± SDs). Different letters indicate a significant difference between samples at $p < 0.05$.

(a)

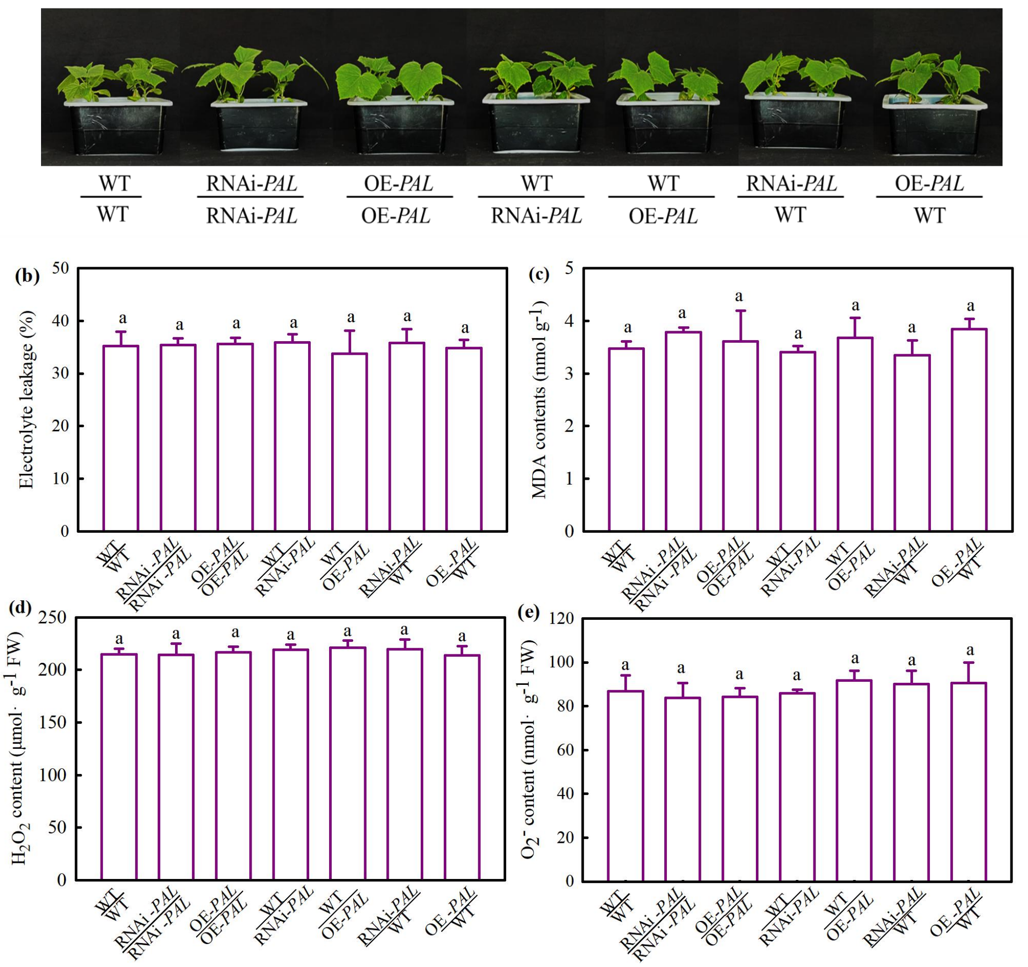

Fig. S4 Effect of RNAi and overexpress *CsPAL* on the cold tolerance of self-grafted and grafted cucumbers at 25/18 °C. **(a),** Phenotype of seedlings; **(b-e),** EL, MDA, H_2O_2 and $O_2^{\cdot -}$ content, respectively. Data are the average values of four biological replicates (±SDs). Different letters are significantly different between samples at $P < 0.05$.

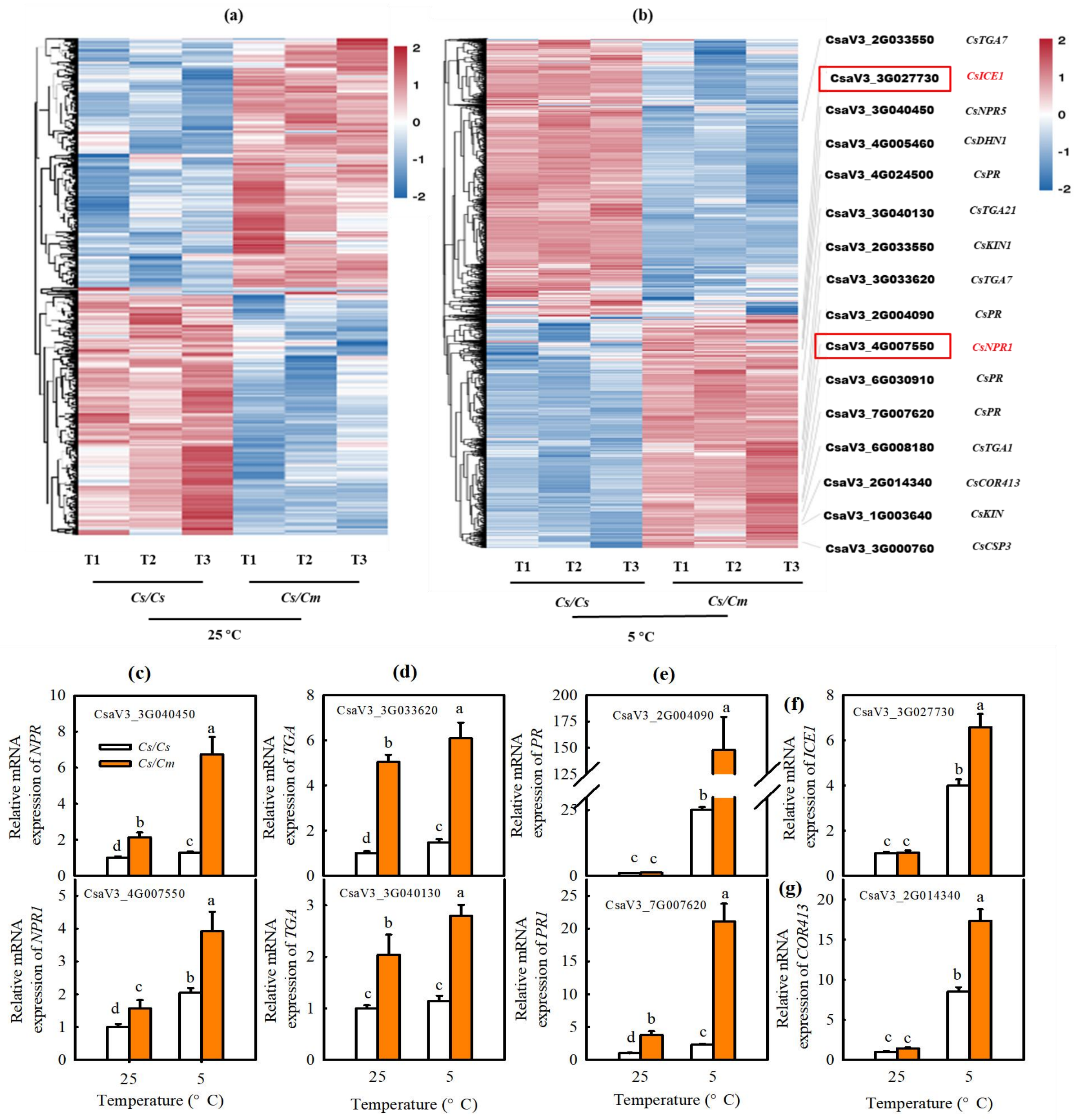

Fig. S5 RNA-seq analysis of *Cs/Cs* and *Cs/Cm* plants at 5 °C or 25 °C for 12 h. **(a, b),** DGEs heat map at 25 ℃ or 5 ℃, respectively; **(c-g),** Relative mRNA expression of *CsNPR, CsTGA, CsPR, CsICE1,* and *CsCOR413*, respectively at 25 °C or 5 °C for 12 h. Data were presented as the mean of four biological replicates (± SDs). Different letters indicate a significant difference between samples at $p < 0.05$.

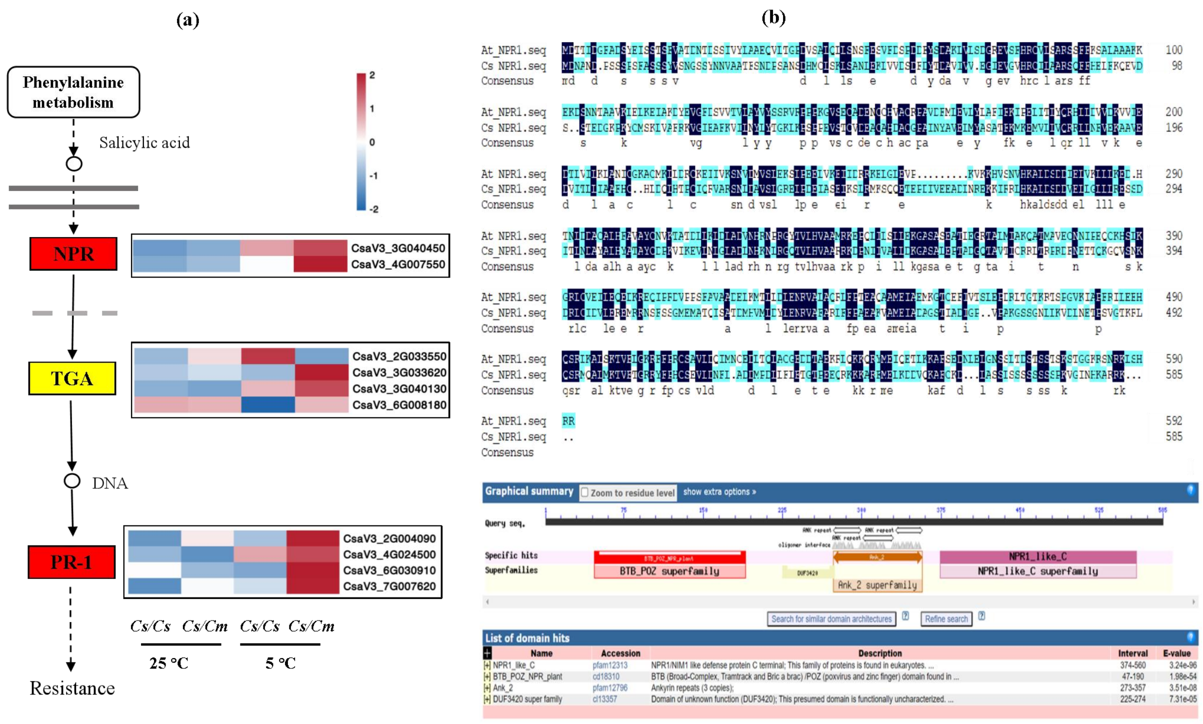

Fig. S6 Gene analysis of *CsNPR1*. **(a),** KEGG pathway map and heat map of the SA signal transduction related DGEs. Red represents up-regulated genes, green represents down-regulated genes, and yellow represents both up-regulated and down-regulated genes; **(b),** Homology alignment of *CsNPR1* and *AtNPR1* amino acid sequence; **(c),** Conserved domain analysis of *CsNPR1*.

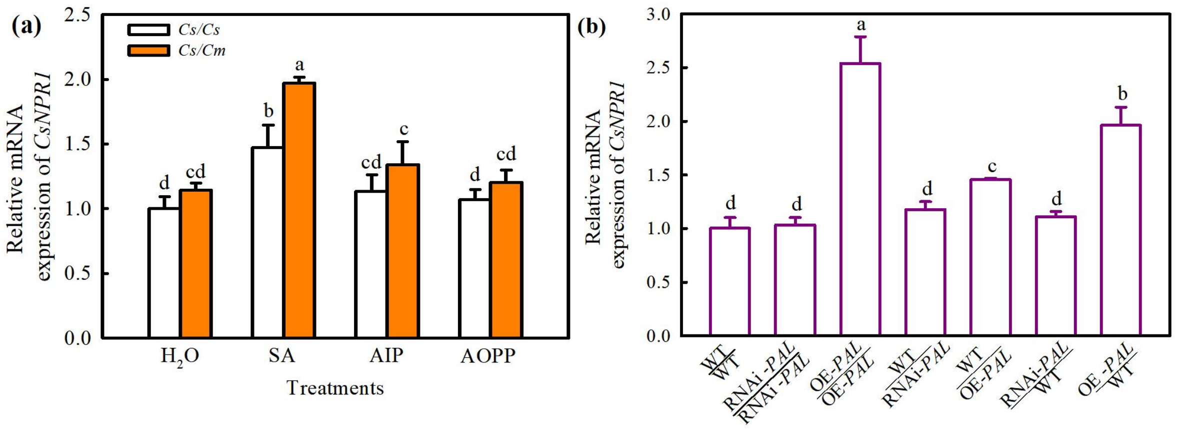

Fig. S7 Effect of exogenous SA and *CsPAL* transgenic on the *CsNPR1* expression of self-grafted and grafted cucumbers at 25/18°C. **(a),** Effect of SA, AIP and AOPP on *CsNPR1* mRNA abundance in *Cs/Cs* and *Cs/Cm* leaves at 25/18°C. **(b),** Relative mRNA expression of *CsNPR1* in *CsPAL* transgenic self-grafted and grafted cucumbers at 25/18°C. Data are the average values of four biological replicates (± SDs). Different letters are significantly different between samples at $P < 0.05$.

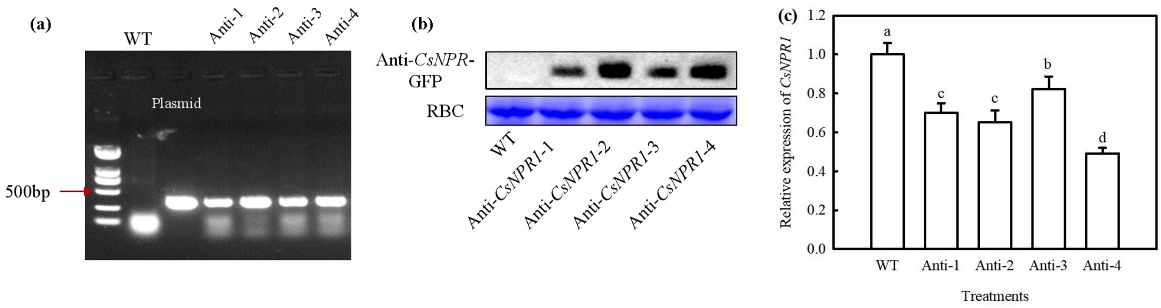

Fig. S8 Identification of Anti-*CsNPR1* transgenic plants. **(a),** PCR electrophoresis results of Anti-*CsNPR1* plants; **(b),** Identification of GFP fusion protein in Anti-*CsNPR1* transgenic plants; **(c),** Relative mRNA expression of *CsNPR1*. Data were presented as the mean of four biological replicates (± SDs). Different letters indicate a significant difference between samples at $p < 0.05$.

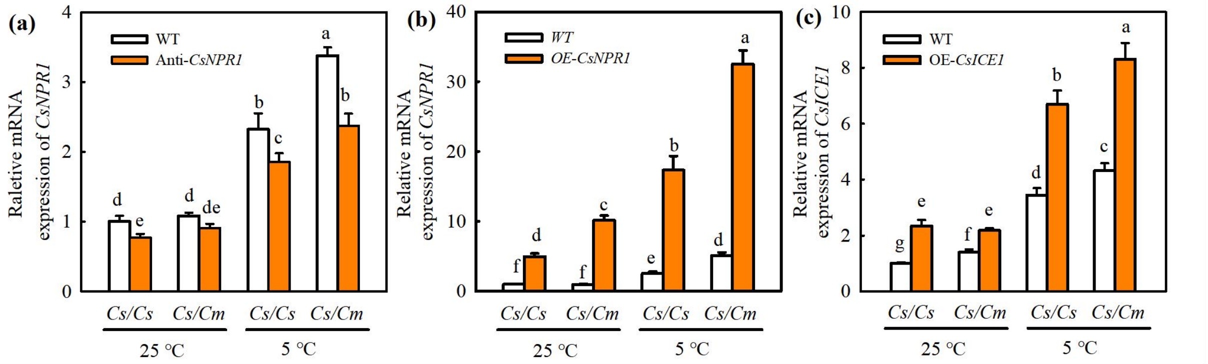

Fig. S9 Relative mRNA expression of *CsNPR1* and *CsICE1* in transient transgenic *Cs/Cs* and *Cs/Cm* cucumber. **(a),** Relative mRNA expression of *CsNPR1* in WT and Anti-*CsNPR1* transgenic plants after treated at 5 °C for 0 and 24 h. **(b),** Relative mRNA expression of *CsNPR1* in empty vector (WT)/*Cs*, *OE-CsNPR1/Cs* and WT/*Cm*, *OE-CsNPR1*/*Cm* transient transgenic cucumber leaves, respectively after treated at 5° C for 0 and 12 h. **(c),** Relative mRNA expression of *CsICE1* in empty vector (WT)/*Cs*, *OE-CsICE1/Cs* and WT/*Cm*, *OE-CsICE1*/*Cm* transient transgenic cucumber leaves, respectively after treated at 5° C for 0 and 12 h. Data were presented as the mean of four biological replicates (± SDs). Different letters indicate a significant difference between samples at $p < 0.05$.

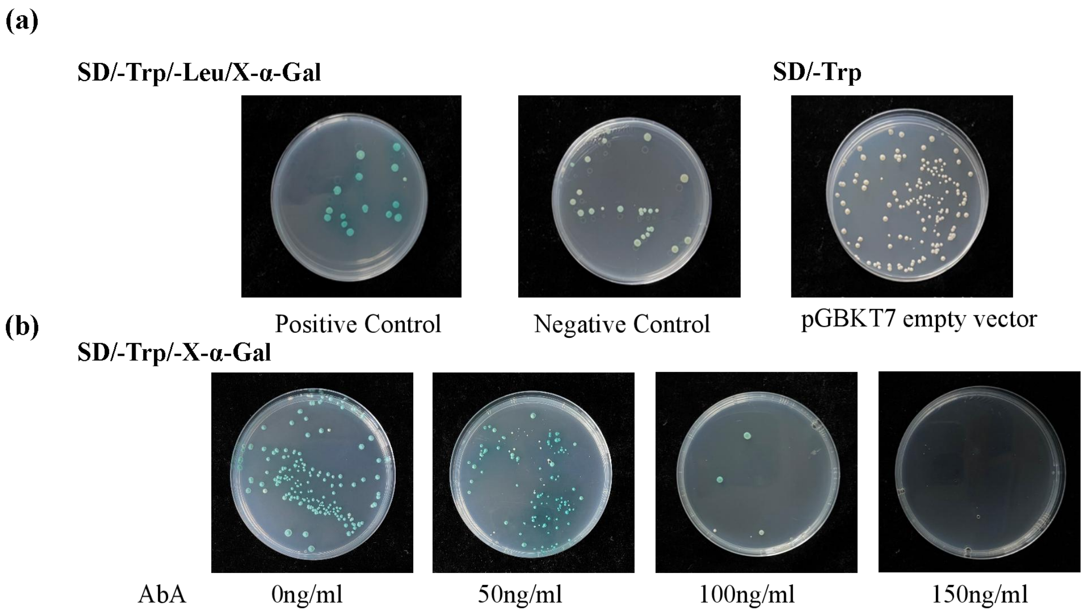

Fig. S10 Identification of CsNPR1-pGBKT7 auto-activation. **(a),** pGBKT7-Lam control vector and pGADT7-T control vector as negative control, pGBKT7-53 control vector and pGADT7-T control vector as positive control were transformed to the yeast cells and plated on SD/-Trp-Leu (SD/-T-L) media and pGBKT7 empty vector was transformed to the yeast cells and plated on SD/-Trp (SD/-T) media. **(b),** The CsNPR1-pGBKT7 yeast cells were plated on SD/-Trp (SD/-T) media with 0, 50, 100, 150 $ng \cdot ml^{-1}$ AbA for 2 d. X-α-gal was used to confirm the activation of reporter genes.

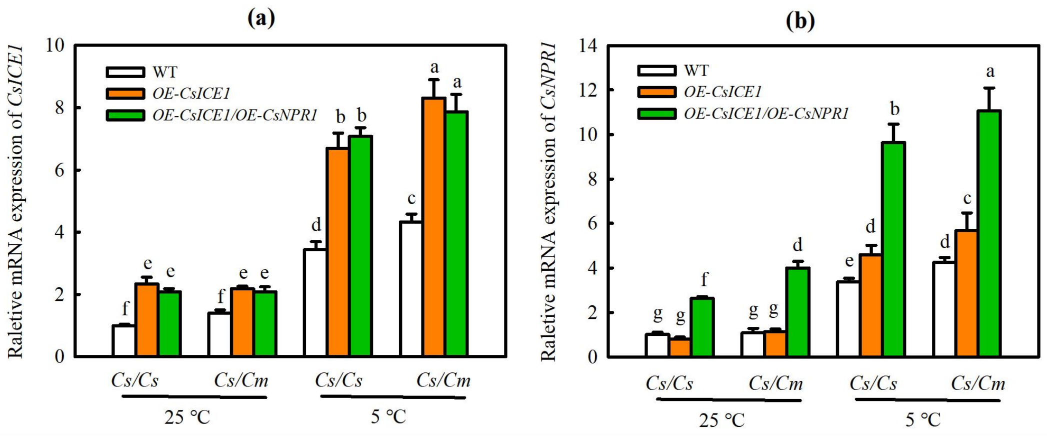

Fig. S11 Co-overexpression of *CsNPR1* and *CsICE1* upregulate the relative mRNA expression of *CsICE1* **(a),** and *CsNPR1* **(b)** in grafted cucumber leaves under chilling stress. The empty vector (WT), OE-*CsICE1* and *OE-CsICE1/OE-CsNPR1* transient transgenic cucumber leaves were treated at 5° C for 0 h and 9 h. Data were presented as the mean of four biological replicates (± SDs). Different letters indicate a significant difference between samples at $p < 0.05$.

Table S1 Primers used for vector construction.

Gene	Primer sequences
PAL-pBI121-Xba Ⅰ F	gagaacacgggggactctagaATGGCCCTAGTCGAGACCATT
PAL-pBI121-Sma Ⅰ R	taagggactgaccacccgggACATATAGGAATTGGAGCACCGTT
RNAi-PAL-tgtF(+):	cagtGGTCTCacaacggaggagtacagaaggcccc
RNAi-PAL-tgtF(-):	cgatGGTCTCacaggccaaatccagtagtaacacc
RNAi-PAL-loop(+):	cgatGGTCTCacctgcaggtctagtttttct
RNAi-PAL-loop(-):	cgatGGTCTCagcccgggctctgtaactatc
RNAi-PAL-tgtR(+):	cagtGGTCTCagggcccaaatccagtagtaacacc
RNAi-PAL-tgtR(-):	cagtGGTCTCatacaggaggagtacagaaggcccc
NPR1-PBI121-Xba Ⅰ -F	gagaacacgggggactctagaATGGACAATGCCAATGATCCC
NPR1-PBI121-Sma Ⅰ -R	ataagggactgaccacccgggTTTTCTCCTAGCTTTATGATTTATACCC
Anti-NPR1-PBI121-Xba Ⅰ -R	gagaacacgggggactctagaAACAAAATTTAGGAGTCGACGCTG
Anti-NPR1-PBI121-Sma Ⅰ -F	ataagggactgaccacccgggAAACTAAGTGCAAACCTCGAGAAGC
ICE1- PBI121 -Xba Ⅰ -F	gagaacacgggggactctagaATGCTTCCCAAATCCACCG
ICE1- PBI121- Sma Ⅰ -R	ataagggactgaccacccgggTACAACACCATGTAAACCAGCTGA
NPR1-BD-EcoR Ⅰ -F	atggccatggaggccgaattcATGGACAATGCCAATGATCCC
NPR1-BD-Pst Ⅰ -R	ctagttatgcggccgctgcagGGATGACAAACTTGACGCTAAGTC
ICE1-AD-EcoR Ⅰ -F	gccatggaggccagtgaattcATGCTTCCCAAATCCACCG
ICE1-AD-BamH Ⅰ -R	cagctcgagctcgatggatccTACAACACCATGTAAACCAGCTGA
NPR-pET32a-BamH1-F	gccatggctgatatcggatccATGGACAATGCCAATGATCCC
NPR1-pET32a-Sal Ⅰ -R	tgcggccgcaagcttgtcgacTTTTCTCCTAGCTTTATGATTTATACCC
ICE1-pGEX-Xho Ⅰ -F	gaattcccgggtcgactcgagATGCTTCCCAAATCCACCG
ICE1-pGEX-Xho Ⅰ -R	tcagtcacgatgccgctcgagTACAACACCATGTAAACCAGCTGA
NPR1-cLUC-Bgl Ⅱ -F	agcggaggaggaagcagatctATGGACAATGCCAATGATCCC
NPR1-cLUC-Pst Ⅰ -R	atacgaacgaaagctctgcagTTTTCTCCTAGCTTTATGATTTATACCC
ICE1-nLUC-Bgl Ⅱ -F	ccactagtgtcgaccagatctATGCTTCCCAAATCCACCG
ICE1-nLUC-Bgl Ⅱ -R	tcctccacgcgtacgagatctTACAACACCATGTAAACCAGCTGA
NPR1-nYFP-F	atcgaggacgccggcggatcATGGACAATGCCAATGATCCCTCCTC
NPR1-nYFP-R	gctctgcaggtcgactctagTCATTTTCTCCTAGCTTTATGATTTATACCCACTTTGG
ICE1-nYFP-F	attacaggtacccggggatcATGCTTCCCAAATCCACCGGAATCC
ICE1-nYFP-R	gccaccgccgtcgactctagTACAACACCATGTAAACCAGCTGAATCCAAC

Table S2 Primers used for qRT-PCR.

Gene	Accession Number	Primer sequences
CsActin	XM_011659465	5'-AGAAGATCTGGCATCACA-3'<br>5'-TCCAATCCAGACACTGTACT-3'
CsICE1	XM_011653285	5'-CGCATCGAGTTGGCTCTGGTG-3'<br>5'-GTCCTCATCGCCGTTCATCTTCC-3'
CsDREB1A	XM_004140746	5'-TACAGAGGAGTCAGGAGGA-3'<br>5'-AGAATCGGCGAAATTGA-3'
CsCOR47	XM_011659051	5'-TGTTCAAGAGGGTGGTGTCG-3'<br>5'- GGATCGGGTGAGTTTCTCCA -3'
CsDREB1B	XM_004143656	5'-CCTATTTATCGTGGTGTGC-3'<br>5'-AGAGTCAGCAAAATTAAGACAA-3'
CsCOR15	XM_004146607	5'-CTATGCGTATGAAGACGCGAAG-3'<br>5'-CGAGGCCGTCTCTGAGGCTTTG-3'
CsCOR413	XM_004145299	5'-TCGGTGCTTCGGGTTTTACT-3'<br>5'-GACGGAGCACAACAGCAATG-3'
CsKIN1	XM_004152338	5'-GGAGAAGCCAAGGGTCAAGC-3'<br>5'-TCATTTGTTCATTCCGGTGG-3'
CsPAL	XM_004143212	5'-ATGGCTTCATATTGCTCTGAG-3'<br>5'-ATGCCTCAAGTCAATTGCTTG-3'
CsRCA	FJ980456	5'-AAAGTGGGCTGTAGGCGTTG-3'<br>5'-TTTTCTATTGTCATCTTCGGTTGG-3'
CsrbcL	EF208123	5'-GCTATGGAATCGAGCCTGTTG-3'<br>5'-CCAAATACATTACCCACAATGGAAG-3'
CsNPR1- CsaV3_4G007550	XM_004147699	5'-TGGAAGAAGCGGACCTGAAC-3'<br>5'-AGGCTCCCTTGTCCAGAAGA-3'
NPR5-CsaV3_3G040450	XM_004136605	5'-TCTTGGCGGCTAGGAGTTTG-3'<br>5'-GCGGAGAGAGTATGAAGGGC-3'
TGA-CsaV3_3G033620	XM_031881719	5'-GACGACACGAAGGTCGAAGA-3'<br>5'-CGGCCAAAAATCTCACGGTC-3'
TGA-CsaV3_3G040130	XM_011653881	5'-GTCCGAAGTGCAGATGGGTG-3'<br>5'-AGTCCTTGTGCTGGTGTCTG-3'
PR-CsaV3_2G004090	XM_004139191	5'-GTGGACGTTGTTTGAGGGTG-3'<br>5'-GATGGCCTCTCAAGTTTCCA-3'
PR-CsaV3_7G007620	XM_004136876	5'-CGGACCTGTCAGTTGGGATG-3'<br>5'-ACGCACGAGTTTGAGGCATA-3'

Table S3 The trimmed *CsNPR1* sequence insert to pGBKT7.

ATGGACAATGCCAATGATCCCTCCTCGTCTCCAAGTTTTGCCTCGTCGTCTTATGTGTCAAATGG
TTCCAGTTATAACAACGTTGCTGCAACATTCAGCAATGACCCTTCTGCAAATTCTGATCATATGT
GTCTTAGCAAACTAAGTGCAAACCTCGAGAAGCTTGTGGTTGATTCTGATTTTGACTATACCGAT
GCGGTGATTGTTGTTGAGGGCATTGAAGTGGGTGTTCATCGATGTATATTGGCTGCTCGGAGTC
AGTTTTTTCATGAGCTTTTTAAGCAGGAAGTGGATAGCTCCACCGAAGATGGCAAGCCAAAATA
TTGTATGTCTAAGTTGGTGGCTTTCAGAAAGGTTGGAATTGAAGCTTTCAAGGTTATCTTGAATTA
CTTGTACACTGGAAAGCTAAAGCCATCACCGCCAGAAGTATCAACATGTGTGGATGAGGCCTGT
GCTCATGATGCTTGTGGCCCTGCTATTAATTATGCAGTGGAATTGATGTATGCTTCTGCCACTTTC
AAGATGAAAGAGATGGTTTTGCTCGTACAGCGTCGACTCCTAAATTTTGTTGAGAAAGCCGCAG
TGGAAGACGTGATAACTTTACTGATTGCTGCATTTCATTGTCATCTGGACCAGCTGCACACCCCC
TGCATTCAAAGAGTCGCAAGGTCGAACCTTGATGCTGTGTCTCTAGGGAGAGAGCTTCCTGATG
AGATTGCAAGTGAAATTAAATCACTGCGTATGAAATCTCAGCAAGAAACTGAACCAGATATTGT
GGAAGAAGCGGACCTGAACCGTGAGAAGAAAATCAGGAGGCTCCACAAAGCATTGGATTCTG
ATGATGTCGAACTACTGGGACTATTGTTGCGTGAATCGTCCGACATTACCTTGAATGATGCTTAT
GCTCTTCATTATGCCACTGCCTATTGTGACCCTAAGGTCATTAAGGAAGTTCTTAACCTAGGGTT
AGCAGATCTCAACCATAAAAACCTTAGAGGACAAACAGTTCTCCATGTTGCAGCAAGGCGTAA
GGATCCTAATATCATTGTAGCTCTTCTGGACAAGGGAGCCTCTGCATTAGAACCTACAGCTGAT
GGACAAACTGCTGTTACAATCTGTCGAAGACTGACTAGGCCTAGGGACTTTAATGAGACAACTC
AAAAAGGACAGGTATCTAACAAAGATCGGTTATGCATTGATGTGCTGGAGAGAGAGATGCGTA
GGAATTCTTTTTCTAGTGGCATGGAAATGGCAACTCAGATTTCCGCTACCGATATGCATGTGATG
CTAGACTATCTAGAAAATAGAGTGGCCTTTGCTCGCCTTTTTTTCCCTGCCGAGGCGAAGGTGGC
AATGGAAATTGCAGATGCAGGTTCCACAATAGCAGATATTGGCCCTGTACCAGCCAAGGGGTC
ATCTGGCAACTTGCTGAAGGTTGACTTGAATGAAACACCATCTGTTGGAACCAAGAGACTTCAG
TCAAGAATGCAAGCTCTAATGAAAACAGTGGAGACAGGTCGACGGTATTTCCCTCATTGCTCGG
AAGTACTCGACAACTTCTTGGCTGATGACATGCCTGACCTTCTCTTCCTGGAAACGGGAACTCC
AGAAGAGCAGCGGAAGAAGAAGGCTCGGTTCATGGAACTTAAAGATGATGTTCAAAAAGCATT
CTGCAAAGACTTAGCGTCAAGTTTGTCATCC

Table S4 *CsNPR1* specific fragment

AAACTAAGTGCAAACCTCGAGAAGCTTGTGGTTGATTCTGATTTTGACTATACCGATGCGGTGAT
TGTTGTTGAGGGCATTGAAGTGGGTGTTCATCGATGTATATTGGCTGCTCGGAGTCAGTTTTTTC
ATGAGCTTTTTAAGCAGGAAGTGGATAGCTCCACCGAAGATGGCAAGCCAAAATATTGTATGTC
TAAGTTGGTGGCTTTCAGAAAGGTTGGAATTGAAGCTTTCAAGGTTATCTTGAATTACTTGTACA
CTGGAAAGCTAAAGCCATCACCGCCAGAAGTATCAACATGTGTGGATGAGGCCTGTGCTCATG
ATGCTTGTGGCCCTGCTATTAATTATGCAGTGGAATTGATGTATGCTTCTGCCACTTTCAAGATG
AAAGAGATGGTTTTGCTCGTACAGCGTCGACTCCTAAATTTTGTT

Table S5 *CsPAL* specific fragment

CAACGGAGGAGTACAGAAGGCCCCTTGTTAAGCTTGGAGGTGAAACTCTTACTATTTCTCAGGT
GGCTGCCATTGCTACTCGAGATTCTGATGTTATTGTTGAGCTTTCTGAGTCCGCTAGAGTCGGTG
TTAAGGCTAGTAGTGATTGGGTTATGGAGAGCATGAATAAAGGCACTGATAGTTATGGTGTTAC
TACTGGAT
